# Does walking protect against decline in cognitive functioning among breast cancer patients undergoing chemotherapy? Results from a small randomised controlled trial

**DOI:** 10.1371/journal.pone.0206874

**Published:** 2018-11-28

**Authors:** Kajal Gokal, Fehmidah Munir, Samreen Ahmed, Kiran Kancherla, Deborah Wallis

**Affiliations:** 1 School of Sport, Exercise & Health Sciences, Loughborough University, Loughborough, Leicestershire, United Kingdom; 2 National Centre for Sport and Exercise Medicine, Loughborough University, Loughborough, Leicestershire, United Kingdom; 3 Leicester Royal Infirmary, University Hospitals of Leicester, Leicestershire, United Kingdom; TNO, NETHERLANDS

## Abstract

**Background:**

Cancer related cognitive impairments have been subjectively reported and objectively detected in breast cancer patients treated with chemotherapy and are known to have a profound negative impact on productivity, psychosocial well-being and overall quality of life. Moderate levels of walking are known to be of benefit to the psychosocial well-being of those affected by breast cancer and for managing cognitive impairment in healthy adults, children, and the elderly. The purpose of this study is to investigate the effects of a home-based, self-managed, moderate intensity walking intervention on subjective and objective cognitive functioning in breast cancer patients undergoing chemotherapy.

**Methods:**

A home-based, self-managed intervention that consisted of moderate levels of walking was compared to usual care among breast cancer patients treated with chemotherapy in a randomised controlled trial. Outcome measures included changes in subjective (CFQ) and objectively detected cognitive functioning (Stroop, SART and two subscales from the WAIS- Digit Span and Block Design). Fifty participants were randomised to either the intervention group (n = 25), who completed 12 weeks of moderate intensity walking, or to the control group (n = 25) mid-way through chemotherapy.

**Results:**

Compared with the control group, the self-managed walking intervention had positive effects on perceived cognitive function but not on sustained attention, executive function, memory or visual spatial skills when assessed objectively using neuropsychological measures.

**Conclusion:**

This home-based, self-managed intervention is beneficial for protecting against perceived cognitive decline in breast cancer patients treated with chemotherapy. There is a need for further research to objectively assess cognitive decline within this population with larger sample sizes of patients.

**Trial registration:**

Current Controlled Trials ISRCTN50709297

## Introduction

Cancer related cognitive impairments (CRCI) in breast cancer patients and survivors have been subjectively reported [1] and objectively detected [2], [3] as an adverse reaction to chemotherapy. Subjective cognitive function is defined as a patient’s self-perceived experience of mental processes and function [4], whereas objective cognitive function refers to mental processes that are assessed using neuropsychological measures [5]. Evidence suggests that 21–90% of breast cancer patients report difficulties in cognitive function [1] and 15–45% are objectively detected [6]. Chemotherapy can have long-term effects on self-reported and objective cognitive functioning of breast cancer patients, with impairments evident from four months to 20 years post-chemotherapy [7], [8]. CRCI have been objectively detected and subjectively reported in a range of cognitive domains including memory, attention, concentration and executive function [5], [9], [10] and can be subtle or dramatic, temporary or permanent, and stable or progressive [11]. Furthermore, cognitive decline as subjectively reported by cancer survivors can have a profound negative impact on productivity and work ability [12], as well as detrimental effects on patients’ feelings of fatigue, anxiety, depression and overall quality of life [13], [14]. It has been suggested that subjective complaints and objective assessment of CRCI are not associated with one another [15–18] but in fact associated with psychosocial distress and fatigue [19] which may be a predisposing risk factor for developing CRCI following chemotherapy for breast cancer [15–18], [20–24].

Despite growing evidence for subjective and objective CRCI, interventions addressing these difficulties among breast cancer patients both during and following primary treatment for cancer have been limited, varied in methods, and report mixed results. The wide range of intervention methods have included: cognitive behavioural training [25], [26]; cognitive training [27–29]; memory training [30]; and physical activity programs ranging from Tai Chi, yoga and Qigong (posture and breathing) to aerobic and resistance training and walking [31–38]. Mixed effects on objective cognitive function have been found post-treatment in breast cancer *survivors*, through the implementation of cognitive behavioural training [26], memory and health training [30], cognitive training [27], [29] and physical activity [32], [33], [36], [38], [39]. Hartman et al. (2017) noted improvements in processing speed following a 12 week physical activity intervention in their sample of breast cancer survivors who were within 2 years of diagnosis, leading them to suggest the need for early implementation of exercise interventions. Similarly, interventions to manage cognitive difficulties experienced by breast cancer *patients* during chemotherapy have yielded mixed results [28], [31]. Poppelreuter et al. (2009) implemented cognitive training strategies during chemotherapy and found no effects on objective cognitive function, whereas Baumann et al. (2011) found positive effects on cognitive function following 12 weeks of resistance training using a battery of neuropsychological measures.

In contrast, evidence demonstrates promising effects of easy-moderate levels of walking as a protective strategy for managing cognitive impairment in healthy individuals, and the elderly [40], [41] as assessed by objective measures. Greater physical activity has also been associated with better working memory and executive function in breast cancer survivors [19], [42] as detected objectively using neuropsychological measures. In light of the evidence outlined above, we propose that moderate levels of walking may also be of benefit to patients receiving chemotherapy for their breast cancer.

To our knowledge, this is the first intervention to investigate the effects of a self-managed, home based, moderate intensity walking intervention among breast cancer patients receiving chemotherapy.

The primary outcomes for the intervention were changes in subjective and objective cognitive functioning. Secondary outcome measures investigated the effects of the intervention upon the psychosocial functioning (anxiety, depression, fatigue, self-esteem and mood) of breast cancer patients. The results for the secondary outcomes have been published elsewhere [43].

In the current study we hypothesise that a self-managed, home based moderate intensity walking intervention may help in managing CRCI experienced by breast cancer patients during chemotherapy.

## Method

### Design

The randomised controlled parallel trial compared 12 weeks of self-managed moderate intensity walking plus usual care (n = 25) to usual care alone (n = 25). Subjective and objective assessments of cognitive functioning and psychosocial measures were completed pre-intervention and 12 weeks later at post-intervention (the same measures were also completed prior to the experimental sessions, but as this was only for the purpose of familiarisation, these data did not contribute towards the main analyses). Ethical approval for the study was obtained from both Loughborough University and the NHS Research Ethics Committee in East Midlands and Northampton (REC ref: 11/EM/0437, date of REC approval: 02/02/12). All participants provided written consent. Research was conducted according to the principles expressed in the Declaration of Helsinki. At the time of initial participant enrolment, the researchers were unaware of trial registration resulting in a delay in registering the current study. The authors confirm that all ongoing and related trials for this intervention are registered.

### Recruitment

Participants were recruited over a 16-month period from three outpatient clinics at the Leicester Royal Infirmary, UK between 01/06/12 and 01/10/2013. Patients with a diagnosis of breast cancer waiting to begin adjuvant or neo-adjuvant chemotherapy who were considered fit to participate in moderate intensity exercise by their oncologist were invited to take part in the study. The researcher met the participants following their initial consultations with their oncologist, provided them with a participant information sheet and explained the nature of the study. Those who showed an interest were followed up 5–7 days later. Women aged between 18 and 75 years were eligible for the study if they: had a primary diagnosis of stage I to III breast cancer; were waiting to begin chemotherapy; were able to read and speak English; were able to walk unassisted; and were relatively inactive (<30min a day, 5 times a week of moderate intensity walking). They were excluded if they had previously been diagnosed with cancer or if they had a current psychiatric illness that could hinder participation in the intervention.

### Procedure

**Fig 1:**
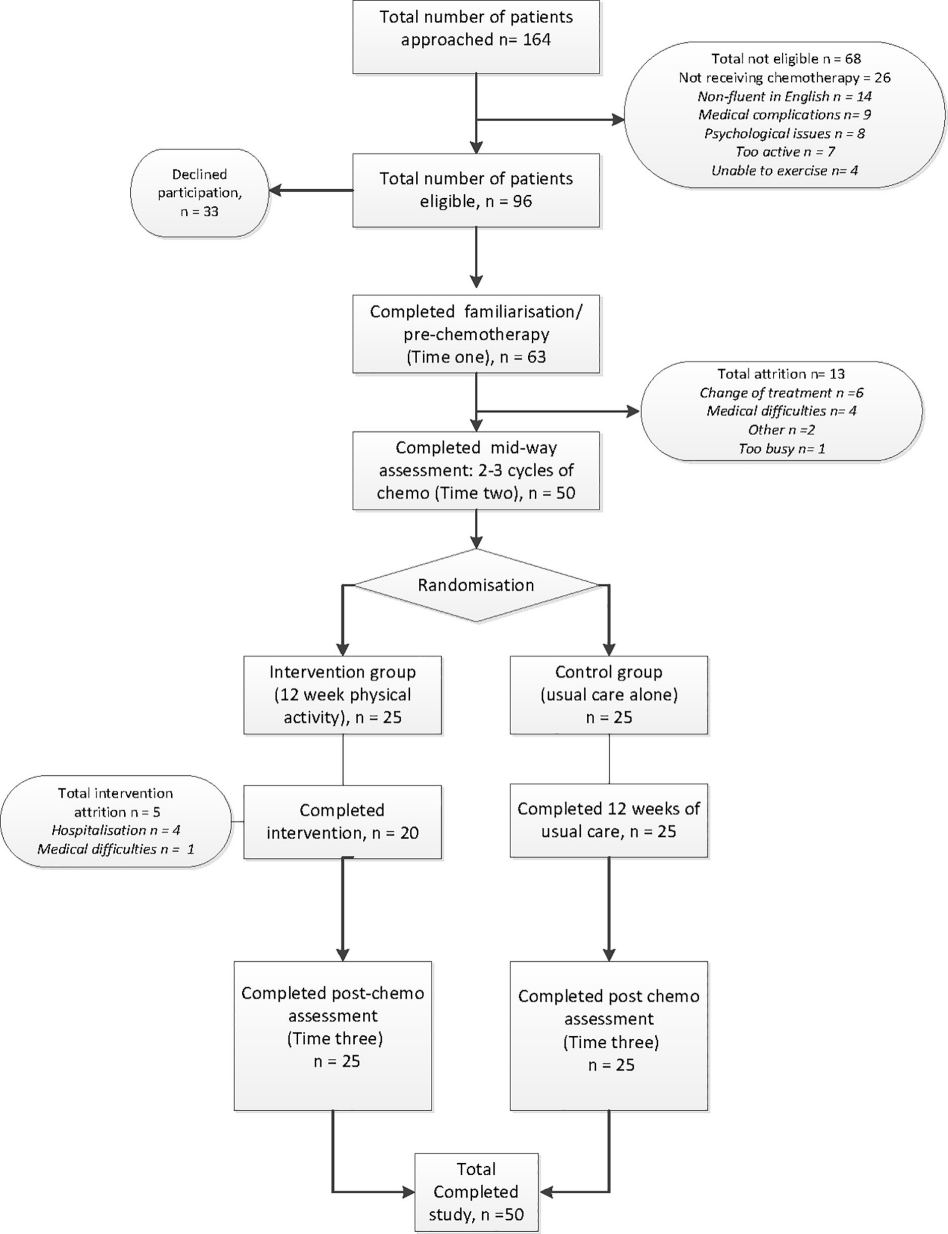
CONSORT Flowchart: Study recruitment and attrition rates.

Cognitive functioning was measured objectively using neuropsychological assessments and subjectively using self-report measures of cognitive difficulties. Measures of Psychosocial well-being and both objective and subjective measures of physical activity were also collected. Experimental assessments took place at two-time points: pre-intervention (after two cycles of chemotherapy) and post-intervention (after the completion of six cycles of chemotherapy). All participants had been prescribed eight cycles of FEC or FEC-T chemotherapy. Participants were randomised to either the intervention (n = 25) or control (n = 25) group after completing the pre-intervention assessment. Following guidance from oncologists, the same measures were also completed pre-chemotherapy to enable familiarisation with the researcher and research methods (see Fig 1). Measures were counterbalanced within each of the cognitive tasks to avoid practice effects.

Participants began the walking intervention after two cycles of treatment, as discussions with oncologists suggested that the intervention would be better received after patients had begun chemotherapy and understood what they were facing. Therefore, assessments of cognitive and psychosocial functioning were compared at time two and time three (pre- and post-intervention) in line with oncologist recommendations. Those who were randomised into the physical activity group were provided with the intervention materials and those in the control group continued with usual medical care alone provided by oncology nurses and doctors. At the time of data collection, patients receiving treatment at the Leicester Royal Infirmary were not routinely advised of the benefits of physical activity during chemotherapy and therefore the control group did not receive any information from either the researcher or their medical team encouraging them to be more active.

### Intervention

The intervention consisted of 12 weeks of home-based, self-managed, moderate intensity walking compared with usual care alone. The design of the walking intervention was based on the Theory of Planned Behaviour (TPB) [44]. Full details of the intervention and materials are reported elsewhere [43], [45]. Patients were provided with an intervention booklet including guidance and recommendations to promote adherence to the intervention, tips and encouragement outlining the benefits of walking, and a copy of the Borg Rating of Perceived Exertion Scale [46] (RPE) which encouraged them to rate the intensity of their walking. They were also provided with a diary to keep a log of walking duration and intensity (using the RPE) and to log their weekly goals based on principles of the TPB. Walking schedules were self-managed; however, the researcher recommended that participants begin by completing 10 minutes of walking at any one time and then steadily increasing the duration to 30 minutes five times a week, in line with recommended guidelines of 150 minutes of moderate to vigorous intensity exercise per week for the general population [47] and breast cancer survivors [48]. Patients were also provided with the researcher’s contact details in case they had any questions regarding the intervention or the booklet and were encouraged to discuss any potential side effects with their health professionals should they occur.

The intervention group was provided with a Yamax Digi-Walker SW-200 pedometer for the duration of the intervention to measure daily step count, to provide patients with immediate feedback, and with the aim of enhancing motivation for the 12-week period. They were also asked to keep a daily exercise diary including the number of steps taken, duration of walking bouts and perceived exertion rates. Those randomised to the control group continued to receive usual care alone.

### Measures

Demographic information was gathered via a recruitment questionnaire and disease or treatment related data was gathered via medical records. Assessments of psychosocial well-being included anxiety and depression (Hospital Anxiety and Depression Scale) [49], mood (Profile of Mood States) [50], fatigue (Functional Assessment of Cancer Therapy- Fatigue) [51], and self-esteem (The Self-Esteem Scale) [52]. Further details about psychosocial measures can be found elsewhere [43], [45]. Measures of cognitive functioning and physical activity are described below. Self-reported physical activity and subjectively and objectively measured cognitive function were conducted at familiarisation, pre and post intervention.

### Objective measures of cognitive functioning

#### Executive function

A computerised version of the Stroop task [53] was used to measure executive function in two blocks. The first required participants to name, using a key press, the print colour of a series of four Xs, and the second consisted of colour words printed in incongruent ink colours (e.g. the word ‘red’ printed in blue). The incongruent condition requires inhibition of the pre-potent response of responding to word meaning. Reading the colour name occurs as an automatic cognitive sub-routine, which interferes with the recognition of the colour itself. In each of the two blocks, 96 stimuli were presented (24 presentations of four colours: red, green, blue, and yellow) and in the incongruent task each colour word was presented six times in each of the four colours. The task was counterbalanced each time it was completed (familiarisation, time two, and time three). Mean reaction time was recorded for each of the two tasks, following the removal of outliers +/- 2 standard deviation’s (SD’s), and an interference (difference) score was calculated to give a measure of executive functioning. This task was chosen as it has frequently been used to illustrate CRCI in chemotherapy patients [13], [23], [54], [55].

#### Working memory

Working memory was assessed using forwards and backwards digit span as used in the Wechsler Adult Intelligence Scale-III [56]. Each task is made up of six pairs of numbers which were read aloud by the researcher. The ‘digits forward’ version specifically targets the phonological loop and requires participants to repeat number sequences in the order in which they are presented. ‘Digits backwards’ targets the visuospatial sketchpad and central executive processes of working memory by asking participants to repeat the number sequence in reverse order, therefore making the task more challenging. Scoring was paper-based with scores for digits forward ranging from 3–9 and digits backwards ranging from 2–8 with high scores in both tasks indicating better performance. Digit span is commonly used to demonstrate CRCI in breast cancer patients [13], [23], [54], [55], [57], [58]. A meta-analysis of the sensitivity of neuropsychological tests used to detect CRCI in breast cancer patients [59] found that digit span produced the largest effect size out of all the tests they reviewed.

#### Attention

Sustained attention was measured using a computerised version of the Sustained Attention to Response Task (SART) [60]. The task presented participants with sequences of digits between 1 and 9 in a quasi-random order and varying font size at a rhythmic rate of one every 250 milliseconds. There were 225 stimuli (each of the 9 digits displayed 25 times), each followed by a mask displayed for 900 milliseconds. The mask is a ring with a diagonal cross inside which acts as a distracter to break up the presentation of the digits. Participants were asked to press the same response key each time, which rapidly becomes an automatic response. However, they were required to withhold this automatic response when they were presented with the number ‘3’. The number of false presses (responses to the number ‘3’) and reaction times before and after false presses were recorded. The SART has previously demonstrated lower scores of sustained attention in cancer patients who had higher levels of emotional distress following diagnosis in comparison to patients who were more emotionally stable at this same time point [61].

#### Perceptual organisation

Visuospatial skills were measured using the WAIS Block Design as used in the Wechsler Adult Intelligence Scale-III [56]. This task required participants to arrange a set of coloured blocks in the same pattern as that demonstrated by the researcher or shown in picture format within the specified time limit. This becomes increasingly difficult through the addition of blocks and the complexity of the designs presented. This test was selected as in a previous meta-analysis [59] it demonstrated significant moderate effect sizes of sensitivity in determining CRCI in breast cancer patients.

#### Self-reported cognitive functioning

The Cognitive Failures Questionnaire (CFQ) [62] measured subjective cognitive functioning on a 25 item scale focusing on minor mistakes made across a one month time frame. This self-report measure generates a score between 1 and 100, with higher scores indicating higher levels of subjective cognitive failures. The scale takes approximately five minutes to complete and has frequently been used with breast cancer patients [16], [21], [54], [63–65].

### Physical activity

#### Accelerometer

All participants were provided with ActiGraph GT3X+ accelerometers and were instructed to wear the device for 10 hours a day for seven days at baseline and after the completion of chemotherapy. In line with previous findings, seven days of continuous monitoring is recommended to assess habitual physical activity in adults and provides a trade-off between feasibility, reliability and acceptable participant burden [66]. Accelerometers were clipped to clothing or worn on a belt above the hip and measured the frequency, intensity and duration of physical activity assessed through body movement.

#### Pedometer

The Yamax Digi-walker SW-200 pedometer was worn by participants randomised to the intervention group for 10 hours a day for the duration of the 12-week intervention. The devices were attached to participants’ clothing above the hip and recorded the number of steps taken per day. Participants were asked to make a daily note of steps taken before resetting the device.

#### Exertion

The Borg Rating of Perceived Exertion Scale [46] was completed by participants in the intervention group to measure the intensity of walking exercises. Subjective measures of perceived exertion are often measured using the RPE in this population [67–70]. The scale asked participants to rate how hard they feel their bodies are working based on the physical sensations they experience, including increased heart rate, breathing rate and sweating. Exertion is measured on a rating scale between ‘6’ and ‘20’ with ‘6’ indicating ‘no exertion at all’ and ‘20’ indicating ‘maximal exertion’. Moderate intensity exercise is rated between 12 and 14 on Borg’s scale. Patients in the intervention group were asked to rate the exertion of their walking sessions and record it in their walking diaries. They were encouraged to aim towards walking at a moderate intensity.

The Talk Test [71] was used by participants within the intervention group as a guide to monitor the intensity and pace of their walking. This informal subjective measure allows individuals to judge their own intensity with the understanding that if they are carrying out moderate intensity walking they should still be able to maintain a conversation but not sing (whereas carrying out vigorous intensity exercise will prevent individuals from speaking more than a couple of words). The Talk Test was verbally explained to all participants in the intervention group and it was also outlined in the booklets. Participants in the intervention group were encouraged to use the measure whilst carrying out their walking exercises to gauge the intensity of their walking and ensure that they were walking at moderate intensities. As this is an informal measure for personal use by participants no data were recorded. However, it provided participants with immediate feedback regarding the intensity of their walking.

#### Levels of physical activity

The General Practice Physical Activity Questionnaire is a validated tool published by the Department of Health in 2002 to assess physical activity levels in 16–74 year olds. The questionnaire rates physical activity on four levels: active, moderately active, moderately inactive, and inactive. The measure was used to gain subjective measures of physical activity levels pre and post intervention.

### Randomisation

Block randomisation using four blocks was used to allocate patients into one of two groups. Within each group of four patients, two were allocated to the intervention group and two to the control group, and the allocation of groups within each block was random. This method was used rather than simple random allocation, to ensure equal numbers of consecutive patients in both groups, as recruitment was staggered [72].

### Sample size

Sample size calculations were based previous research with this population [25], [26]. The study was designed to detect a standardised effect size of 0.5 [73] for repeated measures ANOVA with a power of 0.80 and two-tailed α set at 5% significance level. Thus, 26 participants were needed per arm. To allow for attrition, 62 participants (31 in each arm) were planned for recruitment. Over the recruitment period, a total of 63 participants completed time one measures. However, 13 were lost due to attrition before randomisation and therefore a total of 50 participants were split between the intervention and control arms.

### Statistical analysis

All analyses were carried out using IBM SPSS version 21.0 for Windows. All between-group differences in categorical variables were analysed using Pearson’s chi-square. Initial analyses compared baseline ratings of subjective and objective outcomes of cognitive function.

Intention to treat (ITT) [74] analysis was used to include all randomised patients in the groups to which they were randomly assigned regardless of subsequent withdrawal from treatment or deviation from the protocol. Mixed model ANOVAs were used to test the difference between the two groups (intervention and control) and difference within each group (pre and post) on outcome measures of cognitive function. Scores at time one and time two were compared for the control and intervention groups using t-tests to follow-up on significant interactions. Lastly, Pearson’s correlations were conducted to explore the relationship between variables of psychosocial well-being and self-reported cognitive function.

## Results

Of the 96 eligible participants 33 (34%) declined participation due to high levels of distress following their diagnosis. In total, 63 breast cancer patients due to begin adjuvant and neo adjuvant chemotherapy met the inclusion criteria, consented to take part, and completed recruitment measures (recruitment rate of 69%). A further 13 participants were lost to attrition, due to changes in treatment as a result of chemotherapy related side effects, after providing consent. Therefore, 50 participants receiving chemotherapy were randomised to the intervention (n = 25) or control group (n = 25) see Fig 1.

### Sample characteristics

The age of participants in the intervention group ranged from 27–74 years (mean = 52.1 years; SD = 11.7) and 29–66 years in the control group (mean = 52.4; SD = 8.9). The majority of participants received adjuvant chemotherapy: 20 (80%) in the intervention group and 21 (84%) in the control group (see Table 1).

**Table 1:** Demographic and treatment characteristics for intervention and control group.

Characteristic	Intervention (n = 25)		Control (n = 25)		
**Age** (years) M (SD)	52.08 (11.7)		52.36 (8.9)		p = .500
BMI M (SD)	27.20 (4.82)		28.25 (5.83)		p = .501
	*N*	*%*	*N*	*%*	*x^2^*
**Education**					
None	3	12	6	24	
GCSE (or equivalent)	11	44	9	36	
A level (or equivalent)	3	12	7	28	.279
Degree	5	20	2	8	
Higher Degree	3	12	1	4	
**Marital status**					
Single	2	8	3	12	
Married/living with partner	19	76	19	76	
Separated/divorced	3	12	2	8	
Widowed	1	4	1	4	.940
**Employment status**					
Working	5	20	5	20	
Sick leave	17	68	16	64	
Retired	3	12	4	16	.917
**Breast cancer type**					
Invasive ductal	24	96	23	92	
Invasive lobular	1	4	2	8	.552
**Cancer grade**					
I	0	0	1	4	
II	5	20	8	32	
III	20	80	16	64	.344
**Chemotherapy type**					
FEC	12	48	9	36	
FEC-T	13	52	16	64	.390
**Treatment type**					
Adjuvant	20	80	21	84	
Neo-adjuvant	5	20	4	16	.713
**Surgery type**					
Lumpectomy	17	68	15	60	
Mastectomy	7	28	10	40	
Segmental	1	4	0	0	.437
**Menopausal status**					
Pre-menopausal	12	48	7	28	
Post-menopausal	13	52	18	72	.150
**Self-report physical activity**					
Inactive	16	64	15	60	
Moderately inactive	4	16	4	16	
Moderately active	5	20	6	24	
Active	0	0	0	0	.940

*Note*. FEC (fluorouracil, epirubicin and cyclophosphamide); FET-T (FEC followed by taxotere).

### Baseline characteristics

Chi-Square analysis revealed no significant differences in self-reported levels of physical activity between groups at pre-intervention *x^2^* (2, N = 50) = 0.12, p = 0.94 (findings published elsewhere [43]). Sixty four percent of the intervention group classed themselves as ‘inactive’ compared to 60% in the control group. There were no significant between group differences in baseline measures using neuropsychological tests in the domains of sustained attention, executive function, memory and visuospatial skills. The intervention group had significantly lower baseline scores on the measure of perceived cognitive function in comparison to the control group; however, when within group effects was entered as a factor this difference was accounted for within the analysis.

There was no significant difference in age between those who completed the study (mean = 52 years; SD = 10.29) and those who withdrew (mean = 55 years; SD = 12.67) following familiarisation. Those who withdrew from the study had lower educational qualifications and were less likely to be in employment. Participants did not differ on any other demographic or cancer-related characteristics (breast cancer type & grade, chemotherapy, treatment and surgery type and menopausal status)- please refer to [43].

### Adherence to the walking intervention

Adherence for the intervention group was calculated based upon the completion of the 12-week physical activity intervention, completion of intervention diaries, and goal setting. Although adherence was not calculated based on total amount of physical activity completed (as walking schedules were self-prescribed by individuals), the duration, intensity and frequency of physical activity completed across the 12-week intervention is reported below. Twenty (80%) out of the twenty-five participants who were randomised to the physical activity group adhered to the intervention and completed walking diaries through recording of goal setting, duration, intensity and frequency of their walking. Five participants discontinued participation within the first few weeks of the 12-week intervention, did not complete diaries, but completed all follow up measures post-intervention. Reasons for discontinuing participation in the intervention included hospitalisation or medical complications.

Of the 20 participants that continued with the intervention, 16 completed walking diaries on a weekly basis and four had one or more weeks of missing data due to hospitalisation but continued with the intervention after they were discharged. Walking schedules were self-prescribed, but it was recommended that participants should aim to walk for 30 minutes, five times a week at moderate intensity. Analysis of weekly walking diaries revealed that the 20 participants who completed the intervention walked at moderate intensity (as recorded using the RPE) for an average of 157.4 minutes per week across the 12-week intervention. On average, patients participated in 4.85 walking sessions per week for an average of 30.49 minutes. Findings suggest that the 20 participants who adhered to the intervention met the recommended guidelines of 30 minutes of moderate intensity walking 5 times a week. There was no significant difference between week one and week 12 of the intervention in the number of minutes walked *F* (1, 19) = 0.14, *p* = 0.71 or the number of sessions completed *F* (1, 19) = 0.03, *p* = 0.85. Findings indicate that patients were able to complete the recommended dose of physical activity throughout the course of the intervention and their chemotherapy treatment.

Findings revealed that participants walked an average of 36,217 steps per week as gathered using the Yamax Digi-walker SW-200. There was no significant difference in the number of steps recorded between week one and week 12 of the intervention *F* (1, 19) = 2.13, *p* = 0.16, suggesting that levels of walking remained consistent across the 12-week period.

### Effects of intervention on physical activity

#### Subjective

Chi-Square analysis showed that at post-intervention significant differences were observed between groups on perceived levels of physical activity, *x^2^* (3, N = 50) = 17.15, p = 0.001. When looking at groups separately, the majority of the intervention group (36%) classed themselves as ‘active’ compared with 0% in the control group (please refer to table of result published elsewhere [45]. Those who received the physical activity intervention altered their levels of perceived physical activity from ‘inactive’ to ‘active’. Furthermore, the majority of the control group remained in the inactive group across the 12-week period. Findings indicate a positive change in subjective levels of physical activity following the 12-week intervention.

#### Objective

Comparisons between objective measures of physical activity between groups were not possible due to low compliance of wearing and returning accelerometers. Thirty-one participants (49%) out of the 63 who completed the familiarisation session at baseline (pre-chemotherapy), returned accelerometers. A total of 32 patients were lost due to non-compliance. The most common reasons for missing data were forgetting to wear the device and forgetting to return the device to the researcher. Out of the 31 participants who returned data, six participants complied with recommended wear time with an average of five days, and average wear per day ranged from 39 minutes to 7 hours. Wear time for the 25 participants that did not comply ranged from 7 hours to 3 days.

Participants were asked to wear the device for a further 7 days post-intervention, after the completion of their chemotherapy. Of the 50 participants randomised, seven participants in the intervention arm and three in the control group returned accelerometers at post-intervention. The most common reasons for missing data at post-intervention were forgetting to wear the device, wearing the device incorrectly preventing valid data collection, declining to wear the device, or not returning the device. Due to low numbers and non-compliance, wear time analysis between groups across the intervention period were not possible. At post-intervention, Chi-square analyses showed no significant differences between the intervention and control group in the proportion of patients forgetting to wear the accelerometer, wearing the device incorrectly preventing valid data collection, declining to wear the device, or not returning the device, x^2^ (3, N = 40) = 2.41, p = 0.49.

### Effect of physical activity intervention on cognitive functioning

Analysis of objectively measured cognitive performance revealed no significant main effects for between or within group, or a significant interaction between the two for Stroop interference, sustained attention, visuospatial skills, or memory as assessed by the digit backwards task. There was also no significant interaction for the digit span forwards task. Additional analyses were conducted to examine the main effects whilst removing the interaction as this can mask a significant main effect. There were small effect sizes for the non-significant interactions effects for all measures of objective cognitive functioning. Therefore, these non-significant findings could be due to an inadequate sample size. However, there were significant main effects between groups *F* (1, 48) = 8.27, *p* < 0.01, *ƞp^2^ =* 0.147 and within groups, *F* (1, 48) = 4.55, *p* = 0.03, *ƞp^2^ =* 0.087. Inspection of the means indicated that digit span was slightly, though significantly, greater in the intervention group (mean = 7.8, SD = 1.21) than the control group (mean = 6.8, SD = 1.09) and at post-intervention (mean = 7.4, SD = 1.24) compared with pre-intervention (mean = 7.0, SD = 1.18).

Analysis of self-reported cognitive failures revealed a significant interaction, *F* (1, 48) = 3.90; *p* = 0.05, *ƞp^2^ =* 0.075. As seen in Table 2, although scores remained stable in the intervention group across the 12 week period, t (24) = -1.26, *p* = 0.9, they increased significantly in the control group, t (24) = -2.39, *p* = 0.02, suggesting that the walking intervention protected against self-reported cognitive decline. Inspection of the means indicated that self-reported cognitive failures were significantly higher in the control group (mean = 45.4, SD = 17.3) than the intervention group (mean = 32.7, SD = 8.4) post-intervention.

**Table 2:** Mixed model ANOVAs to test the difference between the two groups (intervention and control) and difference within each group (pre and post) on outcome measures of cognitive function.

**Variable**	**Pre-intervention**		**Post-intervention**			
	*Intervention (n = 25)*	*Control (n = 25)*	*Intervention (n = 25)*	*Control (n = 25)*		
**Perceived cognitive functioning** CFQ	32.48 (7.05)	39.20 (10.12)	32.68 (8.36)	45.44 (17.35)	**Test of Time x Group Interaction**	
					F = 3.90	
					**p = 0.05**	
					*ƞp^2^ =* 0.075	
					**Test of Main Effects**	
					**Between-participant effects**	**Within-participant effects**
					**(Group)**	**(Time)**
**Sustained attention**						
Errors of omission	7.60 (4.53)	8.96 (4.43)	6.56 (3.35)	9.08 (4.41)	F = 3.18	F = 0.88
					p = 0.81	p = 0.35
					*ƞp^2^ =* 0.062	*ƞp^2^ =* 0.018
Correct	396.95 (55.25)	378.62 (89.99)	407.59 (35.02)	386.22 (97.92)	F = 1.01	F = 1.57
					p = 0.31	p = 0.21
					*ƞp^2^ =* 0.021	*ƞp^2^ =* 0.032
Incorrect	364.65 (54.21)	352.32 (83.83)	357.69 (80.38)	353.47 (68.22)	F = 0.21	F = 0.08
					p = 0.64	p = 0.76
					*ƞp^2^ =* 0.004	*ƞp^2^ =* 0.002
**Executive Function**						
Stroop Interference	145.87 (203.85)	219.18 (190.20)	118.93 (125.98)	177.08 (172.71)	F = 2.17	F = 2.78
					p = 0.15	p = 0.10
					*ƞp^2^ =* 0.042	*ƞp^2^ =* 0.055
**Memory**						
Digit forwards	7.32 (1.37)	6.68 (0.85)	7.84 (1.21)	6.81 (1.09)	F = 8.27	F = 4.58
					p<0.001**	p = 0.03*
					*ƞp^2^ =* 0.147	*ƞp^2^ =* 0.087
Digit backwards	5.56 (1.32)	5.13 (1.54)	5.21 (1.71)	4.88 (1.45)	F = 1.03	F = 1.91
					p = 0.84	p = 0.173
					*ƞp^2^ =* 0.022	*ƞp^2^ =* 0.040
**Visual Spatial Skills**						
Block Design	36.58 (10.77)	35.52 (18.75)	34.83 (12.38)	36.26 (8.67)	F = 0.00	F = 0.76
					p = 0.95	p = 0.78
					*ƞp^2^*<0.01	*ƞp^2^ =* 0.002

M, Mean, SD, standard deviation

*p<0.05

**p<0.01.

a. Welch’s F (assumption of homogeneity of variance violated).

### Exploratory analysis between self-reported cognitive function, psychosocial well-being and physical activity

Further exploratory analysis was conducted in order to examine if any psychosocial wellbeing variables contributed towards self-reported cognitive failures. Change scores from pre- to post-intervention were calculated, and correlations conducted for each group between change in reported cognitive failures and change in depression, anxiety, self-esteem, fatigue, and mood; however no significant associations were observed. Similarly, correlations conducted for each group between change in reported cognitive function and objectively detected cognitive function were non-significant.

Exploratory analyses were conducted using Pearson’s two-tailed correlations to explore the relationship between the amount of self-reported physical activity completed by the intervention group and subjective cognitive function. These revealed a significant negative correlation between mean duration of walking (in minutes) and self-reported cognitive failures, *r*_*s*_ = -0.40, p = 0.05, suggesting that as physical activity increased, self-reported cognitive failures decreased.

## Discussion

This randomised controlled trial is the first to investigate moderate levels of a self-managed, home-based walking intervention on cognitive functioning of patients during chemotherapy treatment for their breast cancer. The aim of the study was to assess the effectiveness of a self-managed, home-based walking intervention on subjectively reported and objectively assessed cognitive function during chemotherapy. There was no effect of the intervention on neuropsychological measures of cognitive function. However, small effect sizes for all non-significant interactions for objective measures of cognitive function suggest that these could be explained by the small sample size. We found that perceived cognitive impairment (as measured by self-reported cognitive failures) remained stable in the intervention group whereas it declined in the control group, indicating that moderate levels of walking may help to protect against decline in self-reported cognitive functioning. The maintenance of subjectively perceived cognitive function in the intervention group provides further support for previous literature reporting the benefits of physical activity such as Tai Chi [39] and Qigong [36] in breast cancer patients on improving self-reported cognitive function. Patients are aware of perceived cognitive impairments and the negative impact they can place on overall quality of life, therefore the maintenance of perceived cognitive functioning is noteworthy as it has important implications for the overall health of patients during treatment.

Exploratory correlations between change in reported cognitive failures and change in depression, anxiety, self-esteem, fatigue, and mood were non-significant. Contrary to predictions, maintenance of perceived cognitive function was not due to improvements in psychosocial well-being following the completion of the home-based moderate intensity walking intervention but may be due to a direct effect of participating in the self-managed walking intervention.

Furthermore, duration of walking completed by patients was negatively associated with self-reported cognitive function, suggesting that as physical activity increased, self-reported cognitive failures decreased, and that improvements in subjectively detected cognitive function might be dose-dependent. The dose-dependent relationship between duration of physical activity and perceived cognitive function make vital contributions to limited literature within this area of research, leading to crucial implications for the healthcare of breast cancer patients treated with chemotherapy.

As previously reported [43], breast cancer patients completing 12 weeks of moderate intensity walking had better psychosocial functioning in comparison to usual care alone. Therefore, this suggests that improvements in psychosocial well-being are not a direct reflection of the amount of physical activity completed but rather the result of patients participating in physical activity. Findings from our study do not support previous findings which suggest a direct relationship between the amount of physical activity completed and psychosocial well-being in breast cancer survivors treated with chemotherapy [75]. Inconsistencies between our study and previous findings may be explained by the self-prescribed nature of our study. Our findings suggest that rather than imposing pressure to conform to a prescribed dose of exercise, successful outcomes may be achieved with self-prescribed levels of physical activity. This notion is further supported by a study investigating the effects of prescribed doses of exercise in depressed patients [76] which reported that completing preferred levels of physical activity produced better psychological and social outcomes in comparison to those completing interventions with prescribed doses of exercise.

To the authors’ knowledge, this is the first intervention to investigate the effects of a self-managed, home-based, walking intervention *during* chemotherapy for breast cancer and makes vital contributions to current evidence and clinical practice. This self-managed intervention requires very little input from health professionals and therefore has the potential to mitigate impairments in perceived cognitive function for a large number of breast cancer patients’ receiving chemotherapy. However, as it is the first intervention of its kind and has a relatively small sample size, as highlighted by small effect sizes for all non-significant measures of objective cognitive function, further research with larger sample size is required to confirm findings. Future interventions could examine the dose and intensity of physical activity required for effective maintenance of self-reported cognitive function.

Contrary to previous research, there was no effect of the intervention on neuropsychological measures assessing sustained attention, executive function, memory and visuospatial skills. However, as the effect sizes for non-significant interactions were small, this suggests that further research with larger sample sizes would help to clarify these findings. Moderate levels of exercise have demonstrated positive effects on cognitive function among breast cancer patients [31], [42], healthy adults, and the elderly [41], [77]. In line with the current study, 20 minute bouts of moderate intensity walking have previously been found to reduce the risk of developing Alzheimer’s disease in adults [41]. Moderate to vigorous amounts of physical activity among breast cancer survivors has also been associated with better executive function and working memory [19], [42]. Therefore, findings from the current study were unexpected, as it was anticipated that similar benefits would be seen among our sample of breast cancer patients.

A possible explanation for null findings of objective cognitive function in the current study may be the intensity and dose of physical activity completed in our small sample of patients. As discussed above, evidence suggest that moderate levels of physical activity can help to improve cognitive functioning [19], [31], [41], [42]. However, unfortunately, due to the lack of valid data collected through accelerometers as an objective measure of physical activity, in the current study we are unable to objectively determine if patients were completing the intensity of physical activity required to observe improvements in neuropsychological measures of cognitive function. Furthermore, although the self-perception of activity levels changed from ‘inactive’ to ‘active’ in the intervention group following 12 weeks of walking, data collected through diaries suggested that overall there was no increase in physical activity over the 12-week period. Therefore, it is possible that the amount and levels of physical activity completed by our sample were not effective in eliciting changes in objective cognitive function and is a limitation of the current study.

A further possible explanation for not detecting an effect of the intervention on objectively measured cognitive function may be due to the selection of neuropsychological measures and the subtle nature of cognitive difficulties experienced by breast cancer patients. Evidence suggests that many standard neuropsychological tests do not detect subtle change experienced by cancer patients [9]. The neuropsychological measures included in the study were selected as in previous research they have successfully detected chemotherapy-induced cognitive decline in breast cancer patients. However, it may be that the selected tests are not sensitive enough to pick up on subtle differences experienced by patients across the 12-week intervention period. A review by the International Cognition and Cancer Task Force [78] has proposed the use of the Hopkins Verbal Learning Test-Revised, Trail Making Test and the Controlled Oral Word Association [79]. Unfortunately, the recommendation to use these tests was not published at the time of designing the current study but may be a valuable addition for future studies to detect subtle cognitive deficits experienced by breast cancer patients during chemotherapy.

In the present study there were no significant effects relating to the backwards digit span task, whilst there was a small but significant improvement in recall on the forwards task from pre- to post-intervention, and superior performance overall for the intervention group compared with the control. It is unclear why our findings differed for the forwards and backwards tasks. However, there is evidence that different strategies are employed during forwards and backwards recall. One argument suggests that, unlike forwards recall, backwards recall involves executive control and may be considered as a complex span measure of working memory [80]. Similarly, the improvements in the forwards task could be explained by the simple recall of digits forwards as opposed to the complex nature of recalling digits backwards which require the use of executive function and attention. Therefore, this is consistent with the null findings observed in the other complex measures of cognitive functioning used in the present study. Mixed measures analyses comparing objective levels of physical activity between groups at pre- and post-intervention were not possible due to low compliance of wearing and returning accelerometers. This is a limitation of the current study but provides vital contributions to research within this vulnerable population. It is anticipated that low compliance was largely the result of the timing in which participants were asked to wear the device. Baseline data was collected in the period between initial consultations with oncologists informing participants that they would receive chemotherapy and beginning treatment. This is a highly distressing time for patients when many are still coming to terms with their diagnosis and are preparing for chemotherapy both physically and emotionally. Follow up measures of objective levels of physical activity were gathered post-intervention when patients had completed treatment and were no longer visiting the hospital.

Previous home-based studies have successfully measured objective levels of physical activity using pedometers or accelerometers, [81–83] and therefore our lack of valid data is surprising. However, these studies were conducted with cancer survivors post-treatment. For breast cancer patients facing treatments other validated measures worn on the wrist or thigh, which were not available at the time of this study, may provide more convenient and accurate collection of objective physical activity. Furthermore, direct interaction with health care professionals in previous studies may have acted as motivation to wear the device for the recommended 7 days. Future self-managed, home-based studies assessing objective levels of physical activity during treatment should consider daily notifications reminding participants to wear their device.

The 80% adherence rate to the intervention is noteworthy and a strength of the self-managed, home-based intervention in line with previous evidence which reported that over 50% of breast cancer patients prefer to exercise alone [84]. Current findings suggest that the intervention was well-received by breast cancer patients receiving chemotherapy and emphasises the need for more self-managed interventions within this population. Further self-managed intervention studies with larger sample sizes of patients receiving treatment for their breast cancer are required in order to strengthen current findings that moderate levels of physical activity can protect patients from a decline in subjective cognitive function.

Overall, our study reports benefits of a self-managed, home-based, moderate intensity walking intervention upon cognitive function when subjectively reported but not objectively detected. These findings further support existing evidence for the lack of associations between subjective and objective measures of cognitive decline experienced by breast cancer patients [1]. A possible explanation for the discrepancy in the reporting and measuring of cognitive decline in our study could be due to differences in methodological procedures involved in the gathering of data. Objective measures of cognitive function only provide a snapshot of the individual’s levels of functioning at the time of assessment and may not detect decline. On the other hand, self-report measures ask patients to rate their experiences over a period of time, which may result in more accurate levels of reporting. Discrepancies between the reporting and measurement of cognitive decline in our sample further support previous studies [1] and confirms the need to include both objective and subjective measures of assessment to provide a comprehensive understanding of patients’ cognitive function.

Positive findings using self-report measures are noteworthy, as patients are concerned with perceived deficits and the impact it places on their quality of life. Therefore, maintaining self-reported cognitive function is important to patients, even though perceived deficits have a limited association with objective measures. This study makes a vital contribution towards the advancement of current clinical practice to improve the quality of life of patients during treatment. Furthermore, the self-managed, home-based nature of the study requires little input from healthcare professionals and therefore can be implemented to benefit a larger population of patients.

## Conclusion

The self-managed, home-based intervention had a good adherence rate and was successful in protecting against decline in self-reported cognitive difficulties experienced by patients treated with chemotherapy for their breast cancer. Surprisingly, intervention effects were not detected for objective measures of sustained attention, executive function, memory and visual spatial skills in the current study, which could be explained by our small effect sizes. Therefore, further investigations with large sample sizes conducted over multiple sites are required in order to examine the effects of physical activity upon objectively detected cognitive impairment among breast cancer patients. These studies should optimise the use of the standardised set of neuropsychological measures recently acknowledged as suitable for detecting subtle cognitive decline experienced by breast cancer patients [78] and should also provide daily notification reminders to patients to wear their accelerometers in order to optimise the collection of objective measures of physical activity.

## Supporting information

S1 File. Consort checklist (DOC)

S2 File. Protocol (PDF)

S3 File. Proposal (DOCX)

## Abbreviations

CRCI: Cancer related cognitive impairments
ITT: Intention to treat
