# Supplementary material for: Does walking protect against decline in cognitive functioning among breast cancer patients undergoing chemotherapy? Results from a small randomised controlled trial
Source: PLoS One. 2018 Nov 28;13(11):e0206874. doi: 10.1371/journal.pone.0206874 (PMC6261560; doi:10.1371/journal.pone.0206874)
Supplement: S2 File. Protocol — (PDF) [file pone.0206874.s002.pdf]

Gokal et al. BMC Public Health (2015) 15:414
DOI 10.1186/s12889-015-1751-0

BMC Public Health

STUDY PROTOCOL Open Access

Can physical activity help to maintain cognitive functioning and psychosocial well-being among breast cancer patients treated with chemotherapy? A randomised controlled trial: study protocol

Kajal Gokal[1,2*], Fehmidah Munir[1,2], Deborah Wallis[1,2], Samreen Ahmed[3], Ion Boiangiu[3] and Kiran Kancherla[3]

Abstract

Background: Evidence suggests chemotherapy treatment for breast cancer is associated with side effects such as cognitive impairment in domains of memory, attention, concentration and executive function. Cognitive impairments reported by patients have been associated with higher levels of emotional distress. To date, intervention studies to alleviate cognitive impairment associated with chemotherapy have focused on psycho-educational techniques or cognitive training. Studies have not yet considered physical activity as a potential for alleviating cognitive problems. Physical activity interventions are reported to be effective in alleviating emotional distress and fatigue in those with breast cancer. They have also been reported to improve cognitive functioning in the elderly, in those suffering with dementia and in children. We propose that physical activity could also help to alleviate cognitive impairments in women diagnosed with breast cancer. The study has been designed using a recently developed taxonomy of behaviour change techniques to reliably report the content of the intervention to allow future replication.

Method: This study will deliver a home-based moderate intensity walking intervention to women diagnosed with breast cancer mid-way through their chemotherapy treatment and will compare them to patients receiving usual care alone. The primary outcome measure for this intervention is changes in an objective measure of memory assessed using the Digit Span. Secondary outcome measures include: objective measures of executive function; attention; visual spatial skills; self report cognitive function; self-report fatigue; anxiety; depression; mood and self-esteem. As emotional distress has been associated with self-reporting of cognitive problems, this intervention will further test whether emotional distress mediates between the amount of walking undertaken during the intervention period and levels of self-reported cognitive functioning.

Discussion: The development of an effective intervention for preventing difficulties in emotional and cognitive functioning of cancer patients' post-treatment will help to guide health care professionals to improve patients' overall quality of life. It will also provide direction for future research, ultimately to improve the day to day functioning of breast cancer survivors.

Trial Registration: Current Controlled Trials ISRCTN50709297.

Keywords: Intervention, Breast cancer, Chemotherapy, Physical activity, Exercise, Walking, Cognitive function, Emotional distress, Psychosocial well-being

* Correspondence: k.gokal@lboro.ac.uk
[1]School of Sport, Exercise & Health Sciences, Loughborough University, Loughborough, Leicestershire LE11 3TU, UK
[2]National Centre for Sport and Exercise Medicine, Loughborough University, Loughborough, Leicestershire LE11 3TU, UK
Full list of author information is available at the end of the article

© 2015 Gokal et al.; licensee BioMed Central. This is an Open Access article distributed under the terms of the Creative Commons Attribution License (http://creativecommons.org/licenses/by/2.0), which permits unrestricted use, distribution, and reproduction in any medium, provided the original work is properly credited. The Creative Commons Public Domain Dedication waiver (http://creativecommons.org/publicdomain/zero/1.0/) applies to the data made available in this article, unless otherwise stated.

Background

Breast cancer is one of the most frequently diagnosed cancers in the UK [1]. Between 15% to 75% of patients treated by chemotherapy experience problems with their cognitive functioning as well as physical and emotional side effects [2,3]. Multiple cross-sectional and longitudinal studies over the last decade have found associations between chemotherapy and cognitive impairments, both subjectively reported and objectively measured, in a proportion of breast cancer patients and survivors [4-10]. Commonly reported cognitive impairments include difficulties with memory, attention, information-processing speed and executive function [11,12]. Studies suggest that around 71% of patients report problems with memory and 64% report difficulties with concentration [13,14]. In addition, patients receiving chemotherapy were 2.25 times more likely to show cognitive decline following chemotherapy in comparison with the control group [13]. The effects of chemotherapy on cognitive function among breast cancer patients can be long-term with impairments evident between 4 months to 20 years post-treatment [15-19]. Cognitive decline following chemotherapy for breast cancer can have a profound negative impact on patients' overall quality of life [13,15,20,21].

Evidence suggests that cognitive problems are more frequently reported by patients than that objectively measured using neuropsychological assessments. Literature suggests that subjective reporting and objective measurements of cognitive decline are not associated with one another [5,6,9,10,22-27] but both are associated with poorer functional outcomes and psychosocial well-being, which may be a predisposing risk factor for developing cognitive impairment following chemotherapy in those with breast cancer. [5,6,13,20,22,23,25,26,28-34].

The above studies suggest the need for evidence-based interventions to prevent cognitive decline experienced by breast cancer patients during chemotherapy. However, very few interventions have been implemented to address cognitive difficulties and have yielded mixed results. For example, Poppelreuter et al. [35] found no intervention effects through the implementation of cognitive training strategies whereas Von Ah et al. [36], who also used cognitive training, reported improvements in self-reported cognitive functioning, better mental health and quality of life outcomes. Ferguson et al. [37] implemented a cognitive behavioural therapy intervention and found no significant effect on self-reported cognitive function [37]. Schuurs et al. [38] delivered a psycho-education programme on cognitive strategies by using goal setting and problem solving techniques. They found positive improvements in the management of objective and subjective cognitive function, psychosocial distress and social functioning in patients with a range of cancer diagnoses [38].

The interventions discussed above were implemented between 12 to 8 months after the completion of treatment. However, evidence suggests that patients report difficulties with cognitive functioning half way through their treatment [39,40]. Evidence suggests that interventions to manage the side effects of chemotherapy would be beneficial to patients mid way through treatment as 75% of patients experience cognitive decline during treatment with 35% of patients continuing to report cognitive problems post-treatment [41]. Therefore, more efforts should be made towards preventing cognitive problems during treatment when it is most prevalent.

The benefits of physical activity among cancer patients are well documented with regards to managing treatment related symptoms such as fatigue, regaining physical functioning, improving quality of life [42,43] and for improving physical functioning and fitness for breast cancer patients [44-51]. A meta-analysis of 28 studies revealed positive effects of exercise on cancer related fatigue both during and after treatment [52]. Physical activity reduced distress and improved fatigue and energy levels at three month follow up [51-53]. Physical activity rehabilitation has also shown to improve levels of anxiety for those affected by breast cancer [54]. Evidence demonstrates promising effects of low to moderate levels of physical activity as a protective strategy for preventing cognitive impairment in healthy individuals, children and the elderly [55-59]. In light of this evidence we hypothesise that moderate levels of physical activity may also help in protecting breast cancer patients against chemotherapy-induced cognitive impairment. Hospital-based and/or supervised interventions can restrict participation as patients undergoing chemotherapy are often busy with treatment schedules, are unwell or have limited access to exercise facilities due to transportation or scheduling difficulties [44]. Therefore, home-based interventions which do not restrict participation may be more feasible and increase adherence.

To summarise, there is good evidence illustrating the benefits of physical activity rehabilitation for cancer patients. However, the effects of physical activity on cognitive functioning for those diagnosed with cancer have not yet been examined. Current studies implementing physical activity as a form of rehabilitation for breast cancer have focused upon the benefits it has on physical fitness, quality of life, fatigue and emotional distress. Very little research has been conducted to explore the effects of physical activity upon cognitive functioning in breast cancer patients. Therefore, the current intervention aims to investigate the effects of a self-prescribed moderate intensity walking on cognitive functioning and emotional well-being of adjuvant and neo-adjuvant breast cancer patients.

Methods

Objectives/hypothesis

The primary aim of the study is to investigate the effectiveness of a home-based moderate intensity walking intervention on reducing cognitive impairment of breast cancer patients receiving chemotherapy. Our primary hypothesis is that women who complete the physical activity intervention will demonstrate less impairment in memory in comparison to women in the non-intervention group. Our secondary hypotheses are that the intervention group will perform better than the control group on objective and subjective measures of cognitive function, and will report better quality of life outcomes than the control group. Furthermore, within the intervention group, emotional well-being will mediate between physical activity level and level of subjectively reported and objectively tested impairment in memory, attention and concentration.

Design

The study is a randomised controlled trial comparing the home based walking intervention group with care as usual control group. Outcomes will be assessed at two time points: mid-way through chemotherapy (pre-intervention) and after the completion of chemotherapy (post-intervention). All participants will also complete outcome measures prior to receiving chemotherapy to familiarise them with study materials, study setting and the researcher.

Recruitment

Sixty two females with a primary diagnosis of breast cancer will be recruited from the Leicester Royal Infirmary (31 participants per group). Patients who have been diagnosed, are waiting to begin adjuvant or neo adjuvant chemotherapy and are considered fit to take part in moderate intensity exercise by their oncologist will be invited to participate in the study by the researcher. Oncologists will subjectively assess patients' suitability for the study based upon their medical history and physical presentation during the consultation. Those interested in participating then meet with the researcher who will provide them with an information sheet and explain the study in further detail. Those participants who show interest are then followed up a week later at which point they are asked to complete a consent form. Participants will be assessed using objective measures of cognitive functioning in a small room near the waiting area. They will also complete a series of self-report questionnaires in the comfort of their own homes and return them using the pre-paid envelope provided.

Inclusion criteria

The researcher will assess patients to ensure they meet the following inclusion criteria: to be female; aged 18–75 at diagnosis; have a primary diagnosis of stage I to III breast cancer; receiving chemotherapy and are able to speak and read English.

Exclusion criteria

Patients will not be eligible for the intervention if they meet the following exclusion criteria: previously diagnosed with cancer; those who currently exceed the recommended guidelines of 30 minutes of moderate intensity exercise five times a week; breast cancer is a secondary diagnosis and have a current psychiatric disorder.

Randomisation

Eligible participants will be block randomised using four blocks to ensure equal numbers of patients in both groups as recruitment will be staggered. Within each group of four patients, two will be allocated to the intervention group and two to the control group at time two (mid way through chemotherapy). There is no masking of participants or the research team.

Intervention

In line with previous literature [60] the intervention will consist of 12 weeks of home-based moderate intensity walking compared with usual care alone. The researcher will provide patients with an intervention booklet which will include: guidance and recommendations to ensure adherence to the intervention; tips and encouragement outlining the benefits of walking; and a diary to keep a log of walking duration and intensity.

In line with recommended guidelines [61], participants will be asked to exercise for 30 minutes five times a week at moderate intensity (defined as walking at a brisk pace). Participants will be encouraged by the researcher to gradually increase walking duration from 10 to 30 minute bouts through the course of the intervention and will be asked to make weekly goals outlining intended exercise intensity, duration and timing (exactly when they intend to exercise). They will be encouraged to reflect upon their achievements or shortfalls from the previous week and to take these into consideration by modifying their goals to ensure they are realistic and achievable. They will also be asked by the researcher to take their chemotherapy schedule into consideration, in order to ensure that they do not over exert themselves or set unrealistic goals during weeks in which they will be receiving treatment. This information will also be presented in the walking booklet. Pedometers will be given to the intervention group at the start of the intervention so that they can be used to measure daily step count and to provide patients with immediate feedback and motivation. Relevant behaviour change taxonomies [62] have been applied to this design in order to allow accurate, reliable replication (Table 1).

Table 1 Definitions of behaviour taxonomy [51] and application to intervention

Behaviour change technique number and label	Definition of BCT	Materials	Summary (use of technique in intervention)
Social support			
1. Social support (unspecified)	Advise on, arrange or provide social support or non-contingent praise or reward for the behaviour.	Intervention booklet	Participants received intervention booklets outlining the benefits of walking during treatment, advising them to increase levels of walking and encouraging them to walk with friends and family.
Feedback and monitoring			
11. Self-monitoring behaviour	Establish a method for the person to monitor and record the behaviour as part of a behaviour change strategy.	Pedometer, intervention booklet & walking diary	Patients were asked to keep a daily record of the number of steps taken alongside the intensity and duration of their walking
Repetition and substitution			
29. Graded tasks	Set easy-to-perform tasks, making them increasingly difficult, but achievable, until behaviour is performed.	Intervention booklet & walking diary	Patients were encouraged to set weekly goals and to gradually increase the number of steps taken week by week. They were asked to challenge themselves with each week but also set realistic/achievable goals whilst taking their chemotherapy schedules into consideration.
Goals and planning			
61. Goal setting (behaviour)	Set or agree to set a goal defined in terms of the behaviour to be achieved.	Walking diary	Patients were asked to set specific weekly step count goals stating when and how many steps they intend to take
65. Action planning	Prompt detailed planning of performance of the behaviour (must include at least one of context, frequency, duration and intensity). Context may be environmental (physical or social) or internal (physical, emotional or cognitive).	Walking diary	Patients were encouraged to set weekly plans outlining the amount of walking they intend to do each day of the week. Action plans included the duration, intensity, day of the week and time of day they aim to complete their walking.
Comparison of outcomes			
72. Persuasive source	Present verbal or visual communication from a credible source in favour of or against the behaviour.	Intervention booklet & verbally	Each patient was presented with an intervention booklet stating the benefits of walking which were adapted from credible sources. The patients' consultant also verbally recommended and encouraged patients to participate in the physical activity involved in the study.

Procedure

The researcher will recruit participants, collect cognitive and psychosocial data at pre and post-intervention and facilitate the delivery of the physical activity intervention. Initially, consenting participants will be asked by the researcher to complete a self-report cognitive functioning questionnaire, a battery of objective tests of cognitive impairment and a questionnaire pack assessing quality of life and emotional well-being pre-chemotherapy to allow familiarisation with the researcher and research materials. They will be asked to wear ActiGraph GT3X+ accelerometers for 10 hours a day for 7 days for a measure of habitual walking after the familiarisation assessment [63]. These data will be collected prior to receiving chemotherapy.

After completing two cycles of chemotherapy, participants will be asked to complete the same measures again at the clinic. Patients will then be randomised to either the intervention or control group following the procedures outlined above. The walking intervention starts after 2 cycles of treatment in line with recommendations from oncologists who suggests that the intervention will be better received by patients after they have had 2 cycles of chemotherapy and understand what they are facing with regard to treatment side effects. The intervention group will be asked to commit to the 12 week walking intervention and will be provided with a booklet outlining the amount of walking they should take part in alongside guidance and tips on how to incorporate this into their daily routines. The intervention group will also be asked to commit to a minimum of 150 minutes of moderate intensity walking per week for the duration of the 12 week intervention, in line with recommended guidelines [61]. They will be told how to monitor their own intensity levels and will be encouraged to aim to walk at moderate levels of intensity for 150 minutes a week for the duration of the intervention. Walking schedules will be self-prescribed

by participants but the researcher will recommend that they begin by completing 10 minutes of walking at any one time and then steadily increase the duration of walking to 30 minutes. Participants will be provided with a pedometer for the duration of the intervention and will be asked to log their walking for the 12 week period. Those randomised to the control group will continue to receive usual care alone.

Post-intervention (after the completion of 6 cycles of chemotherapy), all participants will be asked to wear the accelerometer for a further 10 hours a day for 7 days. In addition, they will be asked to complete the same questionnaires and neuropsychological tests as those completed at pre-chemotherapy and mid way through chemotherapy. At the end of the study the control group will receive the intervention booklet (see Figure 1 for flow chart of study methods).

Measures of physical activity

Accelerometers (motion sensors which detect accelerations produced by the human body), will be worn by all participants for 10 hours a day for 7 days at baseline and post-intervention to assess habitual physical activity [63]. Pedometers will be worn by participants randomised to the intervention group for 10 hours a day for the duration of the twelve week intervention in order to record frequency of steps taken. They will also complete the Borg Rating of Perceived Exertion Scale [64] and The Talk Test [65] to subjectively monitor the intensity of their walking during the intervention.

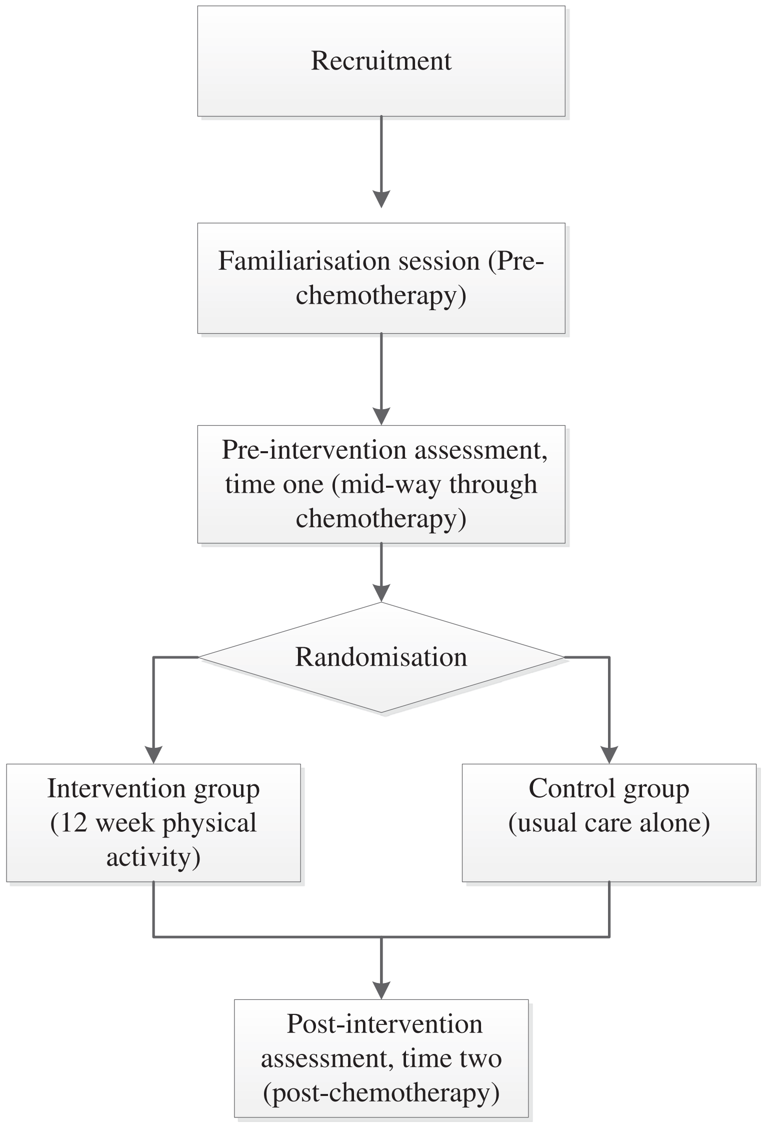

Figure 1 Flow diagram illustrating study methods.

Outcome measures

All outcome measures will be completed at three time points: baseline (familiarisation), mid way through chemotherapy (pre-intervention) and after the completion of chemotherapy (post-intervention). All outcome measures have been validated in this population.

Objective measures of cognitive functioning

The Stroop test [66] will be used to measure executive function. Working memory will be tested using forwards and backwards digit span as used in the Wechsler Adult Intelligence Scale-III [67]. Sustained attention will be measured using a computerised version of the Sustained Attention to Response Task [68]. Visuospatial skills will be measured using the WAIS Block Design subset as used in the Wechsler Adult Intelligence Scale-III [67].

Subjective measure of cognitive functioning

The Cognitive Failures Questionnaire [69] is a 25 item questionnaire which will be used to assess the primary outcome measure of perceived cognitive function. The questionnaire will ask participants to reflect on minor mistakes made across a one month time frame. Responses are measured on a five-point Likert scale ranging from 'very often' to 'never'.

Subjective measures of emotional wellbeing

Anxiety and depression will be measured using the 14 items Hospital Anxiety and Depression Scale [70]. Fatigue will be measured using The Functional Assessment of Cancer therapy-Fatigue [71]. Self esteem will be measured using the Self-Esteem Scale [72]. The rating scale requires participants to indicate how they feel in relation to a series of 10 statements. Emotional Distress will be measured using the shortened version of Profile of Mood States [73]. Participants will first be asked to complete questions regarding their anxiety and depression, followed by self-esteem, fatigue, perceived cognitive function and lastly mood.

Additional information collected at baseline

Demographic and cancer diagnosis data will be collected from all participants at baseline. Participants will be asked to state the average number of hours they spend exercising in an average week before involvement in the study using the General Practioner's Activity Questionnaire.

Sample size

Sample size for the study was calculated based upon previously published data [37,38] and Cohen's Power Primer [74]. According to a prospective calculation, for a repeated measures ANOVA, 26 participants per group are required to detect a large effect size with power of 0.80 and α set at .05. It is reasonable to assume a large effect size in this population based upon previous research [37,38] and their memory performance on the digit span. Allowing for 20% attrition due to withdrawal and illness we aim to recruit 31 patients per group.

Statistical analyses

Quantitative data will be entered into Statistical Package for the Social Sciences (SPSS) and intention to treat analysis will be used in line with the CONSORT statement. To test the effectiveness of the physical activity intervention on cognitive function, a series 2x2 mixed ANCOVAs will be carried out controlling for age and menopausal status (controlling for natural differences in cognitive function associated with age and menopausal status). Participant group (intervention or control) will be entered as the between subjects factor and time (pre-intervention and post-intervention) will be entered as the within subjects factor. In addition, to test the effectiveness of the intervention on quality of life and measures of well-being another series of 2x2 mixed measures ANOVA will be carried out between the intervention group and control group. Post-hoc analysis will be carried out using a series of t-tests to check for differences between the intervention and control group. Lastly, to test whether emotional distress mediates between amount of physical activity (duration of walking and steps taken) and self-report cognitive functioning for the intervention group, a series of mediation analyses will be carried out using regression analyses.

Ethics

Written informed consent will be gained by all patients and GPs will be informed of their participation within the study. The study has been granted ethical approval by the National Health Service, local ethics board for the Leicester Royal Infirmary and by Loughborough University's ethics committee.

Discussion

Cognitive difficulties associated with chemotherapy in adjuvant and neo-adjuvant breast cancer patients have been well established in research but rarely addressed through the implementation of interventions. As the number of breast cancer survivors increases each year research has become more focused upon developing effective interventions to aid transition back to everyday functioning (post-treatment). This intervention has been developed with the aim of managing cognitive difficulties following treatment which often affect patients for years after completion. Cognitive difficulties experienced by patients following the completion of treatment have been reported to challenge their abilities to return to work, in turn lowering levels of self-esteem further.

The outcomes of this intervention will add valuable insight into treatment for cognitive difficulties experienced by breast cancer patients as a result of chemotherapy which is an essential element of their treatment. Findings will provide healthcare professionals with guidance for additional care both during and after the completion of treatment and help improve current services. It will also help direct future research to improve the ongoing well-being of breast cancer survivors.

Competing interests
The study is funded by Loughborough University as part of a PhD project. The authors declare that they have no competing interests.

Authors' contributions
KG, FM and DW contributed to the conception and design of the study as well as writing and editing the manuscript. SA, IB and KK are contributing to the acquisition of data through the Leicester Royal Infirmary Hospital. All authors read and approved the final manuscript.

Acknowledgements
We would like to thank all participants who have agreed to take part in the clinical trial and have given us their time and input into the study. This activity was conducted under the auspices of the National Centre for Sport and Exercise Medicine (NCSEM) England, a collaboration between several universities, NHS trusts and sporting and public bodies. The views expressed are those of the authors and not necessarily those of NCSEM England or the partners involved.

Author details
[1]School of Sport, Exercise & Health Sciences, Loughborough University, Loughborough, Leicestershire LE11 3TU, UK. [2]National Centre for Sport and Exercise Medicine, Loughborough University, Loughborough, Leicestershire LE11 3TU, UK. [3]Leicester Royal Infirmary, Infirmary Square, Leicester LE1 5WW, UK.

Received: 20 October 2013 Accepted: 16 April 2015
Published online: 23 April 2015

References
1. Cancer Research UK. Breast cancer briefsheet. 2008. Retrieved December 4, 2008, from http://publications.cancerresearchuk.org/WebRoot/crukstoredb/444CRUK_PDFs/CRBSBRC08.pdf.
2. Ferguson RJ, Riggs R, Ahles T, Saykin AJ. Management of chemotherapy-related cognitive dysfunction. New York: Springer; 2007.
3. Vardy J, Tannock I. Cognitive function after chemotherapy in adults with solid tumors. Crit Rev Onclol/Hemotol. 2007;63:183–202.
4. Berglund G, Bolund C, Fornander T. Late effects of adjuvant chemotherapy and postoperative radiotherapy on quality of life among breast cancer patients. Eur J Cancer. 1991;27:1075–81.
5. Van Dam FS, Schagen SB, Muller MJ, Boogerd W, Vd Wall E, Droogleever Fortuyn ME, et al. Impairment of cognitive function in women receiving adjuvant treatment for high-risk breast cancer: high-dose versus standard dose-chemotherapy. J Natl Cancer Inst. 1998;90:210–8.
6. Schagen SB, van Dam FS, Muller MJ, Boogerd W, Lindeboom J, Bruning PF. Cognitive deficits after postoperative adjuvant chemotherapy for breast cancer carcinoma. Cancer. 1999;85(3):640–50.
7. Brezden CB, Phillips K-A, Abdolell M, Bunston T, Tannock IF. Cognitive function in breast cancer patients receiving adjuvant chemotherapy. J Clin Oncol. 2000;18(14):2695–701.
8. Schagen SB, Muller MJ, Boogerd W, Rosenbrand RM, van Rhijn D, Rodenhuis S. Late effects of adjuvant chemotherapy on cognitive function: a follow-up study in breast cancer patients. Ann Oncol. 2002;2002(13):1387.
9. Ahles TA, Saykin AJ, Furstenberg CT, Cole B, Mott LA, Skalla K. Neuropsychological impact of standard-dose systemic chemotherapy in long-term survivors of breast cancer and lymphoma. J Clin Oncol. 2002;20(2):485–93.
10. Tchen N, Juffs HG, Downie FP, Qi-Long Y, Hanzian H, Chemerynsky L. Cognitive function, fatigue and menopausal symptoms in women receiving adjuvant chemotherapy for breast cancer. J Clin Oncol. 2003;21:4175–83.
11. Mar Fan HG, Houédé-Tchen N, Yi QL, Chemerynsky I, Downie FP, Sabate K, et al. Fatigue, menopausal symptoms, and cognitive function in women after adjuvant chemotherapy for breast cancer: 1- and 2-year follow-up of a prospective controlled study. J Clin Oncol. 2005;23(31):8025–32.
12. Meyers CA, Perry JR. Cognition and cancer. New York: Cambridge University Press; 2008.
13. Shilling V, Jenkins V. Self-reported cognitive problems in women receiving adjuvant therapy for breast cancer. Eur J Oncol Nurs. 2007;11:6–15.
14. Shilling V, Jenkins V, Morris R, Deutsch G, Bloomfield D. The effects of adjuvant chemotherapy on cognition in woman with breast cancer-preliminary results of an observational longitudinal study. Breast. 2005;14:142–50.
15. Boykoff N, Moieni M, Subramanian SK. Confronting chemobrain: an in-depth look at survivors' reports of impact on work, social networks, and health care response. J Cancer Survi. 2009;3(4):223–32.
16. Fitch MI, Armstrong J, Tsang S. Patients' experiences with cognitive changes after chemotherapy. Can Oncol Nurs J. 2008;18(4):180–92.
17. Hermelink K, Küchenhoff H, Untch M, Bauerfeind I, Lux MP, Bühner M, et al. Two different sides of 'chemobrain': determinants and nondeterminants of self-perceived cognitive dysfunction in a prospective, randomized, multicenter study. Psychooncology. 2010;19(12):1321–8.
18. Myers JS. Chemotherapy-related cognitive impairment: the breast cancer experience. Oncol Nurs Forum. 2012;39(1):E31–40.
19. Reid-Arndt SA, Hsieh C, Perry MC. Neuropsychological functioning and quality of life during the first year after completing chemotherapy for breast cancer. Psychooncology. 2010;19(5):535–44.
20. Jenkins V, Shilling V, Deutsch G. A 3-year prospective study of the effects of adjuvant treatments on cognition in women with early stage breast cancer. Br J Cancer. 2006;94:828–34.
21. Steiner JF, Cavender TA, Main DS, Bradley CJ. Assessing the impact of cancer on work outcomes: what are the research needs? Cancer. 2004;101(8):1703–11.
22. Bender CM, Sereika SM, Berga SL, Vogel VG, Brufsky AM, Paraska KK, et al. Cognitive impairment associated with adjuvant therapy in breast cancer. Psychooncology. 2006;15(5):422–30.
23. Castellon SA, Ganz PA, Bower JE, Petersen L, Abraham L, Greendale GA. Neurocognitive performance in breast cancer survivors exposed to adjuvant chemotherapy and tamoxifen. J Clin Exp Neuropsychol. 2004;26(7):955–69.
24. Donovan KA, Small BJ, Andrykowski MA, Schmitt FA, Munster P, Jacobsen PB. Cognitive functioning after adjuvant chemotherapy and/or radiotherapy for early-stage breast carcinoma. Cancer. 2005;104(11):2499–507.
25. Hermelink K, Untch M, Lux MP, Kreienberg R, Beck T, Bauerfeind I, et al. Cognitive function during neoadjuvant chemotherapy for breast cancer: results of a prospective, multicenter, longitudinal study. Cancer. 2007;109(9):1905–13.
26. Jansen CE, Dodd MJ, Miaskowski CA, Dowling GA, Kramer J. Preliminary results of a longitudinal study of changes in cognitive function in breast cancer patients undergoing chemotherapy with doxorubicin and cyclophosphamide. Psychooncology. 2008;17(12):1189–95.
27. Wefel JS, Lenzi R, Theriault RL, Davis RN, Meyers CA. The cognitive sequelae of standard-dose adjuvant chemotherapy in women with breast carcinoma: results of a prospective, randomized, longitudinal trial. Cancer. 2004;100(11):2292–9.
28. Biglia N, Bounous VE, Malabaila A, Palmisano D, Torta DME, D'Alonzo M, et al. Objective and self-reported cognitive dysfunction in breast cancer women treated with chemotherapy: a prospective study. Eur J Cancer Care (Engl). 2012;21(4):485–92.
29. Schilder CM, Eggens PC, Seynaeve C, Linn SC, Boogerd W, Gundy CM, et al. Neuropsychological functioning in postmenopausal breast cancer patients treated with tamoxifen or exemestane after AC-chemotherapy: cross-sectional findings from the neuropsychological TEAM-side study. Acta Oncol. 2009;48(1):76–85.
30. Weis J, Poppelreuter M, Bartsch HH. Cognitive deficits as long-term side-effects of adjuvant therapy in breast cancer patients: 'subjective' complaints and 'objective' neuropsychological test results. Psychooncology. 2009;18(7):775–82.
31. Cimprich B. Attentional fatigue following breast cancer surgery. Res Nurs Health. 1992;15(3):199–207.

32. Mehlsen M, Pedersen AD, Jensen AB, Zachariae R. No indications of cognitive side-effects in a prospective study of breast cancer patients receiving adjuvant chemotherapy. Psychooncology. 2009;18(3):248–57.
33. Menhert A, Scherwath A, Schirmer L, Schleimer B, Petersen C, Schulz-Kindermann F. The association between neuropsychological impairment self-perceived cognitive deficits, fatigue and health related quality of life in breast cancer survivors following standard adjuvant versus high-dose chemotherapy. Patient Educ Couns. 2007;66:108–18.
34. Vearncombe KJ, Rolfe M, Wright M, Pachana NA, Andrew B, Beadle G. Predictors of cognitive decline after chemotherapy in breast cancer patients. J Int Neuropsychol Soc. 2009;15:951–62.
35. Poppelreuter M, Weis J, Bartsch HH. Effects of specific neuropsychological training programs for breast cancer patients after adjuvant chemotherapy. J Psychol Oncol. 2009;27:274–96.
36. Von Ah D, Carpenter JS, Saykin A, Monahan P, Wu J, Yu M, et al. Advanced cognitive training for breast cancer survivors: a randomized controlled trial. Breast Cancer Res Treatment. 2012;3:799–809.
37. Ferguson RJ, McDonald BC, Rocque MA, Furstenberg CT, Horrigan S, Ahles TA, et al. Development of CBT for chemotherapy-related cognitive change: results of a waitlist control trial. Psychooncology. 2012;21:176–86.
38. Schuurs A, Green HJ. A feasibility study of group cognitive rehabilitation for cancer survivors: enhancing cognitive function and quality of life. Psychooncology. 2013;5:1043–9.
39. Vardy J, Wong K, Yi Q, Park A, Maruff P, Wagner L, et al. Assessing cognitive function in cancer patients. Support Cancer Care. 2006;14:1111–8.
40. Munir F, Kalawsky KEA, Lawrence C, Yarker J, Haslam CO, Ahmed S. Cognitive intervention for breast cancer patients undergoing adjuvant chemotherapy: a needs analysis. Cancer Nurs. 2011;34:385–92.
41. Janelsins MC, Kohli S, Mohile SG, Usuki K, Ahles TA, Morrow GR. An update on cancer- and chemotherapy-related cognitive dysfunction: current status. Semin Oncol. 2011;38(3):431–8.
42. Stevinson C, Lawlor DA, Fox KR. Exercise interventions for cancer patients: systematice review of controlled trials. Cancer Causes Control. 2004;15:1035–56.
43. Galvao DA, Newton RU. Review of exercise intervention studies in cancer patients. J Clin Oncol. 2005;23:899–909.
44. Pinto BM, Frierson GM, Rabin C, Trunzo JJ, Marcus BH. Home-based physical activity intervention for breast cancer patients. J Clin Oncol. 2005;23(15):3577–87.
45. Pinto BM, Slark MM, Maruyama NC, Feder SI. Psychological and fitness changes associated with exercise participation among women with breast cancer. Psychooncology. 2003;12:118–26.
46. Campbell A, Mutire N, Whytee F. A pilot study of a supervised group exercise programme as a rehabilitation treatment for women with breast cancer receiving adjuvant chemotherapy. Eur J Oncol Nurs. 2005;9:56–63.
47. Basen-Engquist K, Taylor CLC, Rosenblum C, Smith MA, Shinn EH, Greisinger A, et al. Randomized pilot test of a lifestyle physical activity intervention for breast cancer survivors. Patient Educ Couns. 2006;64:225–34.
48. Ohira T, Schmitz KH, Ahmed RL, Yee D. Effects of weight training on quality of life of recent breast cancer survivors. Cancer. 2006;2076–2083:9.
49. Courneya KS, Segal RJ, Gelmon K, Reid RD, Mackey JR, Friedenrich CM, et al. Six-month follow-up of patient-rated outcomes in a randomised controlled trial of exercise training during breast cancer chemotherapy. Cancer Epidemiol Biomark Prev. 2007;16:2572–8.
50. Milne HM, Gordon S, Guilfoyle A, Wallman KE, Courneya KS. Association between physical activity and quality of life among western Australian breast cancer survivors. Pyschooncology. 2007;16:1059–68.
51. Fillion L, Gangan P, Leblond F, Gelinas C, Savard J, Dupuis R, et al. A brief intervention for fatigue management in breast cancer survivors. Cancer Nurs. 2008;31:145–59.
52. Cramp F, Daniel J. Exercise for the management of cancer-related fatigue in adults (review). Cochrane Database Syst Rev. 2008;2, CD006145.
53. Mock V, Frangakis C, Davidson NE, Ropka ME, Pickett M, Poniatowski B, et al. Exercise manages fatigue during breast cancer treatment: a randomised controlled trial. Psychooncology. 2005;14:464–77.
54. Courneya KS, Segal RJ, Mackey JR, Gelmon K, Reid RD, Friedenreich CM, et al. Effects of aerobic and resistance exercise in breast cancer patients receiving adjuvant chemotherapy: a multicenter randomised controlled trial. J Clin Oncol. 2007;25:28.
55. Colcombe SJ, Kramer AF, Erickson KI, Scalf P, McAuley E, Cohen NJ, et al. Cardiovascular fitness, cortical plasticity and aging. Proc Natl Acad Sci U S A. 2004;101(9):3316–21.
56. Bixby WR, Spalding TW, Haufler AJ, Deeny SP, Mahlow PT, Zimmerman JB, et al. The unique relation of physical activity to executive function in older men and women. Med Sci Sports Exercise. 2007;39(8):1408–16.
57. Newson RS, Kemps EB. Cardiorespiratory fitness as a predictor of successful cognitive aging. J Clin Exp Neuropsychol. 2006;28:949–67.
58. Van Gelder BM, Tijhaus MA, Kalmijn S, Giampaoli S, Nissinen A, Kromhout D. Physical activity in relation to cognitive decline in elderly men: the FINE study. Neurology. 2004;63(12):2316–21.
59. Barnes DE, Yaffe K, Satariano WA, Tager IB. A longitudinal study of cardiorespiratory fitness and cognitive function in healthy older adults. J Am Geriatr Soc. 2003;51(4):459–65.
60. Mishra SI, Scherer RW, Snyder C, Geigle PM, Berlanstein DR, Topaloglu O. Exercise interventions on health-related quality of life for people with cancer during active treatment. Cochrane Database Syst Rev. 2012;8:8465.
61. Department of Health, Physical Activity, Health Improvement and Prevention. 2004. At least five a week. http://webarchive.nationalarchives.gov.uk/+/dh.gov.uk/en/publicationsandstatistics/publications/publicationspolicyandguidance/dh_4080994.
62. Michie S, Ashford S, Sniehotta FF, Dombrowski SU, French DP. A refined taxonomy of behaviour change techniques to help people change their physical activity and healthy eating behaviours-the CALO-RE taxonomy. Psychol Health. 2011;26(11):1479–98.
63. Corder K, Brage S, Ekelund U. Accelerometers and pedometers: methodology and clinical application. Curr Opin Clin Nutr Metab Care. 2007;10(5):597–603.
64. Borg G. Borg's perceived exertion and pain scales. Champaign, IL: Human Kinetics; 1998.
65. Persinger R, Foster C, Gibson M, Fater DCW, Porcari JP. Consistency of the talk test for exercise prescription. Med Sci Sports Exercise. 2004;36(9):1632–6.
66. Stroop JR. Studies of inference in serial verbal reactions. J Exp Psychol. 1935;18:643–62.
67. Wechler D. WAIS manual. New York: The Psychological Corporation; 1995.
68. Robertson IH, Manly T, Andrade J, Baddeley BT, Yiend J. 'Oops!': Performance correlates of everyday attentional failures in traumatic brain injured and normal subjects. Neuropychologia. 1997;35:747–58.
69. Broadbent DE, Cooper PF, FitzGerald P, Parkes KR. The Cognitive Failures Questionnaire (CFQ) and its correlates. Br J Clin Psychol. 1982;21:1–16.
70. Zigmond AS, Snaith RP. The hospital anxiety and depression scale. Acta Pschiatrica Scandinavica. 1983;67:361–70.
71. Yellen SB, Cella DF, Webster K, Blenowski C, Kaplan E. Measuring fatigue and other anemia-related symptoms with the Functional Assessment of Cancer Therapy (FACT) measuring system. J Pain Symptom Manag. 1997;13(2):63–74.
72. Rosenberg M. Society and the adolescent self-image. Princeton, NJ: Princeton University Press; 1965.
73. Shacham S. A shortened version of the profile of mood states. J Pers Assess. 1983;47:3.
74. Cohen J. A power primer. Psychol Bull. 1992;112(1):115.

Submit your next manuscript to BioMed Central and take full advantage of:

- **Convenient online submission**
- **Thorough peer review**
- **No space constraints or color figure charges**
- **Immediate publication on acceptance**
- **Inclusion in PubMed, CAS, Scopus and Google Scholar**
- **Research which is freely available for redistribution**

Submit your manuscript at
www.biomedcentral.com/submit

BioMed Central
