# Supplementary material for: Does walking protect against decline in cognitive functioning among breast cancer patients undergoing chemotherapy? Results from a small randomised controlled trial
Source: PLoS One. 2018 Nov 28;13(11):e0206874. doi: 10.1371/journal.pone.0206874 (PMC6261560; doi:10.1371/journal.pone.0206874)
Supplement: S3 File. Proposal — (DOCX) [file pone.0206874.s003.docx]

**Does physical activity have an impact on cognitive and emotional functioning**

**in Breast Cancer patients?**

*Kajal Gokal, Fehmidah Munir and Deborah Wallis*

**Introduction**

The following proposal outlines a physical activity intervention aimed at managing cognitive dysfunction as reported and objectively measured in a proportion of breast cancer patients undergoing adjuvant/ neo adjuvant chemotherapy. The intervention aims to investigate the effectiveness of home-based moderate intensity walking in improving levels of self-esteem and psychological well-being whilst reducing emotional distress commonly reported by breast cancer patients. In addition, the study aims to investigate if improvements of cognitive functioning following the physical activity intervention are mediated by improvements to emotional distress and self-esteem.

*Cognitive side effects experienced by breast cancer patients*

Medical advancements in cancer treatments have led to increasing survival rates of cancer patients (Cancer Research UK, 2008). Increasing survival rates have led to the identification of a host of physical, emotional and cognitive side effects in cancer patients.

Breast cancer is the most frequently diagnosed cancer in the UK (Breast Cancer Care, 2010). Following surgery, the majority of breast cancer patients may undergo a range of adjuvant treatments including chemotherapy, radiotherapy and or hormone replacement therapies. It has been estimated that approximately 70% of patients experience treatment related symptoms during adjuvant therapy (Kim et al., 2008). The most commonly reported side effects of chemotherapy reported by breast cancer patients are fatigue, pain, anxiety and depression (So et al., 2009). Chemotherapy patients report feeling very tired and fatigued during and for months after completing treatment. In addition, the thinning and loss of hair as well as dry and sensitive skin can also be very distressing for patients undergoing adjuvant therapy for breast cancer (Breast Cancer Care, 2010).

Alongside physical and emotional side effects, cognitive impairment following adjuvant chemotherapy is a common difficulty evident in a proportion of breast cancer patients. Commonly described cognitive impairments include difficulties with memory, attention, information-processing speed and executive function (Meyers & Perry, 2008). As these concerns are becoming more prominent, research has shifted towards managing these side effects and helping to improve overall quality of life and well-being of breast cancer patients and survivors (Brown et al., 2003).

Cognitive ability of breast cancer patients undergoing adjuvant chemotherapy has been reduced in a number or cross-sectional and prospective studies. Studies have focused predominately on the use of objective neuropsychological assessments and in some cases have combined testing with self-report measures.

Berglund et al. (1991) provided one of the first cross-sectional studies demonstrating an association between adjuvant chemotherapy and cognitive impairment (Please refer to table one for a review of cross-sectional studies demonstrating chemotherapy-induced cognitive impairment). They found that breast cancer patients reported higher levels of cognitive impairment as measured by subjective measures in comparison with a treatment control group. However, Berglund and colleagues have been criticised for their exclusive use of self-report measures and their poor validity. As a result, there was a shift in research towards the use of neuropsychological assessments of cognitive functioning in breast cancer patients. Subsequent cross-sectional studies further demonstrated the relationship between adjuvant chemotherapy and cognitive impairment, with a subset of breast cancer patients scoring lower on neuropsychological tests in comparison with controls (Weineke & Dienst, 1995; van Dam et al., 1998; Schagen et al., 1999; Brezden et al., 2000; Schagen et al., 2002; Ahles et al., 2002; Tchen et al., 2003 and Mehnert et al., 2007).

Weinke and Dienst (1995) reported that seventy five percent of the twenty eight breast cancer patients in their study demonstrated moderate impairments in at least one measure of neuropsychological functioning. Moderate impairments were revealed in areas of attention, mental speed, memory, visuo-spatial ability and motor function when compared to published normative data. They also found that the duration of chemotherapy received by the patient was significantly associated with cognitive impairment, suggesting that patients who received longer periods of chemotherapy scored lower on neuropsychological tests. Van dam et al. (1998) similarly demonstrated a relationship between chemotherapy and cognitive impairment. In their cross sectional study of breast cancer patients receiving either high or standard dose chemotherapy, results showed that 32% of patients receiving high dose chemotherapy were cognitively impaired in comparison with 17% receiving standard dose and 9% in the control group. Individuals who scored two standard deviations below the control were defined as cognitively impaired. The study was one of the first to demonstrate a link between chemotherapy dosage and level of cognitive impairment within the breast cancer population. In addition, high doses of adjuvant chemotherapy were also associated with higher levels of fatigue and depression. The higher the dose of chemotherapy the higher the levels of depression and fatigue as reported using subjective measures.

Further associations between chemotherapy and cognitive impairments were demonstrated by Brezden et al. (2000), who investigated immediate and late effects of adjuvant chemotherapy. They found that those who were currently receiving chemotherapy and those who had finished treatment both reported cognitive impairment, suggesting long term effects of chemotherapy upon breast cancer patients. Within the study 48% of patients receiving chemotherapy displayed difficulties in cognitive domains of memory, attention and concentration whereas 50% of those who had completed treatment displayed difficulties in domains of language and visual motor skills in comparison with 11% of controls. Ahles et al. (2002) also investigated long term effects of cancer survivors who were treated with standard dose chemotherapy. In line with previous evidence those who were treated with chemotherapy had lower scores scored lower on measures of verbal memory and psychomotor functioning as assessed by neuropsychological methods. Similarly Tchen et al. (2003) found that 16% of breast cancer patients displayed cognitive impairments in domains of language, attention, concentration, self-regulation and planning in comparison with 4% of a healthy control group.

The above studies have shown a clear association between adjuvant chemotherapy and cognitive impairment in breast cancer patients; however, these results have been subject to a number of methodological limitations (Bender et al., 2006). Firstly, the range of cognitive impairment reported across studies varies hugely due to inconsistent definitions, lack of homogeneous cut-off points of cognitive impairment and due to variations in measurements used (Pullens et al., 2009). In addition, due to the nature of cross-sectional studies they fail to take baseline measure of cognitive functioning. Baseline measures would allow researchers to make comparisons over time and determine if differences observed are due to chemotherapy alone or if they were present before treatment. The lack of control groups in some studies has led to comparisons of cognitive function against published norms. This raises further concerns regarding validity (Weineke & Dienst, 1995 & Philips et al., 2003). Concerns raised due to the reliability of cross-sectional studies have led towards a shift in research towards prospective longitudinal studies incorporating baseline measures and control groups. One of the earliest prospective studies was conducted by Wefel et al. (2004) who measured cognitive impairment at three time points in their small sample of 18 breast cancer patients (please refer to table to for a review of prospective studies demonstrating chemotherapy-induced cognitive impairment). Thirty three percent of patients demonstrated cognitive impairment at pre-treatment, which later increased to 61% at 6 month assessment. Assessment taken 18 month after the first time point suggested that half of the patients who experienced cognitive impairment showed improvements whereas the other half neither improved nor deteriorated further. This study suggests that cognitive impairments are not due solely to chemotherapy, as 33% of patients presented with impairments before chemotherapy. In contrast, a later study (Shilling et al., 2004) using both objective and subjective measures of cognitive function found that patients receiving chemotherapy were 2.25 times more likely to show cognitive decline following chemotherapy in comparison with control group. In addition, they suggested that subjective measures of cognitive function are a reflection of emotional distress and well-being as opposed to cognitive dysfunction. This notion is further supported by a later study conducted by the same research team in 2007 who found that 71% of patients reported problems with memory and 64% reported difficulties with concentration. However, these relatively high percentages of subjective measures of cognitive impairment were not related to the 30% of patients who objectively demonstrated cognitive decline. They used a battery of neuropsychological measures and structured interviews to investigate the difference between subjective and objective measures of cognitive impairment. They found that participants subjectively reported cognitive difficulties in domains of memory and concentration which were not related to objective cognitive dysfunction but rather to psychological distress and quality of life. Furthermore, 34% of chemotherapy patients who displayed difficulties with memory six months into chemotherapy reduced to 15% 12 months after the final session, suggesting that cognitive effects of chemotherapy may improve over time. However, it is possible that reported side effects do not improve over time but that patients feel less anxious and distressed at follow up assessments as they are reaching the end of treatment. Further studies are required to examine this possible association.

In line with previous evidence Quesnel et al. (2009) reported impairments in verbal fluency and the ability to retrieve memorised information in patients receiving chemotherapy in comparison with healthy matched controls at three month follow up assessments. Similarly, Von ah et al. (2009) also support this concept as they found 17% of breast cancer patients scored significantly lower on objective measures in comparison with individually matched healthy controls.

The association between cognitive functioning and psychological variables following chemotherapy was further investigated by Vearncombe et al. (2009). Seventeen percent of the 136 women who took part in their study demonstrated significant decline in domains of verbal learning and memory, abstract reasoning and motor co-ordination. Declines within these domains were significantly associated with baseline measures of fatigue, depression and well-being. Although effect sizes were small, Vearncombe and colleagues suggest that baseline scores of psychological distress may increase the risk of developing cognitive impairments following chemotherapy. This raises questions as to whether cognitive decline demonstrated by breast cancer patients is solely a consequence of chemotherapy or if it is a result of pre-existing psychological distress caused by diagnosis and surgery. This is further supported by a number of studies (Jenkins et al., 2006; Shilling et al., 2007; Pullens et al., 2009 and Schagen et al., 2009) who found that subjective measures were correlated with negative affect and psychological distress and depression as opposed to cognitive impairment. Pullens et al. (2009) reported that 11 out of the 15 studies they reviewed found no significant relationship between subjective and objective measures of cognitive impairment.

*Rehabilitation to improve cognitive impairment experienced by breast cancer patients*

The above studies suggest the need for evidence-based rehabilitation to improve cognitive functioning within cancer patients undergoing adjuvant chemotherapy. Although evidence suggests a need for such an intervention it has seldom been investigated in breast cancer patients. Current literature tends to focus upon interventions aimed at improving quality of life through primarily targeting physical symptoms and distress caused by the disease (Manos et al., 2008; Doulbeaut et al., 2009; Marcus et al., 2010 and Louiselle et al., 2010). The majority of interventions directed towards breast cancer patients tend to exclude those with cognitive difficulties and very few have targeted cognitive impairments revealed during cancer chemotherapy (Please refer to table 3 for a review of intervention studies).

The lack of evidence based cognitive rehabilitation within current literature may be explained by the magnitude of limitations associated with identifying the prevalence of cognitive impairments within breast cancer patients. Inconsistencies in current literature do not allow valid comparisons and conclusions to be drawn regarding the cause and magnitude of cognitive dysfunction in breast cancer patients. Weis, Poppelreuter and Bartsch, (2009) have explained this as a result of inconsistencies in the design of current studies, characteristics by which patients are compared, variations of treatment type, dosage and definition of cognitive impairment. As outlined above, inconclusive evidence of cognitive deficits as measured by self report measures and neuropsychological tests create further difficulties in drawing conclusion and explaining the underlying cause and prevalence of such deficits (Weis, Poppelreuter and Bartsch, 2008 and Pullens at al., 2009).

Literature suggests Ferguson et al. (2007) were the first to publish promising results of cognitive rehabilitation for breast cancer patients. A sample of 29 women reporting long term complaints of memory and attention problems following the completion of chemotherapy were subjected to Memory and Attention Adaptation Training (MAAT) over a four month period. Participants were provided with a work book outlining compensatory strategies, which were discussed with clinicians during four individual visits and further broken down by telephone contact between sessions. MAAT was broken into four cognitive behavioural components including education on memory and attention, self-awareness training, self-regulation emphasising arousal reduction through relaxation training, and compensatory strategies training. Ferguson and colleagues reported overall improvements in self-reported cognitive functioning, quality of life and neuropsychological test performance following completion of treatment and during two and six month follow ups. However, the authors of the study recognised methodological limitations of their study. Firstly, results cannot be generalised due to a limited sample size that were narrowly selected from highly educated Caucasian backgrounds which are not representative of the wider breast cancer population. In addition, the single arm study failed to recruit a control group and as a result we are unable to conclude that the reported improvements are a due to MAAT.

Poppelreuter, Weis and Batsch (2009) recruited a sample of 96 breast cancer patients who were divided into one of three groups; neuropsychological training group (NPT), computer based training (PC) or control group. Both intervention groups were devised to improve functional attention and memory deficits. Patients’ levels of cognitive functioning were measured at three time points: upon admission to the rehabilitation unit, at the end of in-patient rehabilitation and 6 months later. Poppelreuter and colleagues reported no specific intervention effects and noted that the majority of patients demonstrated significant improvement in all three groups as measured by a comprehensive neuropsychological test battery. The lack of consistent positive effects of cognitive interventions for breast cancer patients suggests the need to develop an intervention which targets both the emotional and cognitive side effects of chemotherapy experienced by breast cancer patients.

*Evidence outlining benefits of physical activity rehabilitation for cancer patients*

A growing number of studies and clinical trials have illustrated the benefits of physical activity for cancer patients in regards to managing treatment related symptoms such as fatigue, regaining physical functioning and improving quality of life (Stevinson et al., 2004 and Galvao & Newton 2005). Physical activity and psychosocial factors such as reduced quality of life and emotional distress, which are commonly reported as troublesome for breast cancer patients and survivors, have been successfully improved with physical activity rehabilitation.

A number of studies have demonstrated the positive effects of physical activity in improving physical functioning and fitness for breast cancer patients (Pinto et al., 2003; Pinto et al., 2005; Campbell et al., 2005; Basen-Enguist et al., 2006; Ohira et al., 2006; Courneya et al., 2007a; Milne et al., 2007 and Fillion et al., 2008). Basen-Enguist et al. (2006) randomised 60 breast cancer survivors to either a lifestyle physical activity intervention or standard care control group. Those who formed the intervention group were asked to attend a total of twenty one, 90 minute group based exercise sessions. The intervention group showed promise in the ability to improve endurance and physical aspects of quality of life and to change physical activity in comparison with controls.

A number of studies have demonstrated a positive change in quality of life following physical activity interventions within the breast cancer population (Segal et al., 2001; Courneya et al., 2003; Campbell et al., 2005; Sandel et al., 2005; Cho et al., 2006; Ohira et al., 2006; Daley et al., 2007 and Milne et al., 2007). The above studies have demonstrated an overall increase in quality of life as a result of the implementation of physical activities such as walking, cycling, aerobics and resistance training and muscle strengthening. Quality of life has been typically measured using self-report subjective measures such as the Functional Assessment of Cancer Therapy-Breast. Physical activity interventions showing such changes in quality of life have varied in duration from 4 weeks (Fillion et al., 2008) to 18 weeks (Sandel et al., 2005) and have been delivered in both individual (Courneya et al., 2007) and group settings (Mutrie et al., 2007). Furthermore, in some studies physical activity interventions were delivered alongside telephone contact providing patients with the opportunity to discuss barriers, difficulties and achievements to improve self-esteem and maintain emotional distress whilst enabling facilitators with the opportunity to reinforce efforts and adherence (Segal et al., 2001; Pinto et al., 2005 Milne et al., 2007). Telephone contacts at regular intervals have also been included within physical activity rehabilitation to maintain the interest of patients during intervention.

Positive effects of physical activity have been reported for increasing self-esteem (Courneya et al., 2003, 2007a & 2007b and Pinto et al. 2005). Pinto et al. (2005) found positive effects of home based moderate intensity walking in their sample of 86 sedentary breast cancer patients. The 12 week programme involved women walking for 10 minutes two times a week to begin with and gradually increasing walking duration to 30 minutes. Pinto and colleagues found that the intervention group showed improvements in physiological measures, fatigue and self-esteem. Courneya et al. (2007b) found aerobic and resistance training exercises had long term effects on self-esteem and late effects on anxiety in their sample of stage I to III breast cancer patients. In addition, physical activity rehabilitation has revealed positive effects for managing fatigue (Mock et al., 2005; Pinto et al., 2005; Milne et al., 2007 and Fillion et al., 2008). Milne and colleagues randomised a sample of 29 breast cancer patients, within two years of completing adjuvant therapy, to an exercise intervention group. They found encouraging effects of individual based aerobic and resistance training programme on fatigue as measured by the Schwartz Cancer Fatigue Scale. Those randomised to the exercise group demonstrated a significant decrease in fatigue from baseline to week six of the intervention and a smaller yet significant decrease from six to twelve weeks and 12 to 18 weeks. Whereas, those randomised to control group did not demonstrate significant decrease in levels of fatigue. The combined aerobic and resistance exercise programme was also effective in improving overall quality of physical fitness. In addition, a meta-analysis of 28 studies revealed positive effects of exercise on cancer related fatigue both during and after treatment for cancer (Cramp & Daniel, 2010).

Similarly, Mock et al., (1994) and Fillion et al., (2008) found positive effects of physical activity interventions in reducing distress in breast cancer patients. Fillion and colleagues randomised a sample of 87 breast cancer patients who had completed treatment to either a group exercise intervention or a control group. Patients assigned to the intervention group were asked to take part in weekly group sessions of physical activity and psycho-education plus supervised walking training and fatigue management sessions. Those in the intervention group demonstrated significant improvements in distress at three month follow up in comparison with the control group. Furthermore, participants receiving the physical exercise intervention demonstrated improvements in fatigue and energy levels at three month follow up. Positive effects of physical activity rehabilitation have also shown to improve levels of anxiety for those affected by breast cancer (Courneya et al., 2007b). Courneya and colleagues found that individual-based aerobic and resistance training exercises in patients initiating adjuvant chemotherapy had positive late effects on anxiety in their sample of 160 breast cancer patients. A systematic review of 14 studies looking at the effects of exercise on breast cancer patients and survivors concluded that exercise is an effective intervention to improve quality of life, physical functioning and fatigue (McNeely et al., 2006). The vast array of literature demonstrating the benefits of physical exercise for cancer patients, as outlined above, has led general practitioners and other health care professionals to shift the focus of treatment care. Patients were previously advised to rest and take time out and rest during treatment, however patients are now encouraged to exercise and keep active. Macmillan cancer support similarly encourage patients to stay active both during and after treatment and as a result have developed guidance outlining the benefits of physical exercise along with directions on how to incorporate exercise into their daily routines around treatment (Macmillan Cancer Support, 2011).

*Components of physical activity interventions*

A number of the above studies focused on hospital-based or supervised interventions which do not allow generalisations to patients who have limited access to exercise facilities as a result of transportation or scheduling difficulties (Pinto et al., 2005). To overcome such restrictions some researchers have moved towards the use of moderate intensity home-based physical activity interventions (Pinto et al., 2005; Mock et al., 1997; Courneya et al., 2003 and Yang et al., 2010). A randomised control trial of stage 0-II breast cancer patients who had completed adjuvant therapy found positive effects of a home based physical activity intervention in improving physical activity and psychological well-being (Pinto et al., 2005). Mock et al. (1997) and Yang et al. (2010) also found positive effects of home-based walking interventions in improving mood, emotional distress and quality of life following adjuvant therapy for breast cancer. The incorporation of moderate intensity walking exercises into rehabilitation for breast cancer patients has proven to be successful for improving physical side effects such as reduced physical fitness and fatigue as well as psychological factors such quality of life, emotional distress and mood (Fillion et al., 2008; Mock et al., 2005; Pinto et al., 2003 and Yang et al., 2010). In addition, literature suggests individuals affected by breast cancer prefer moderate intensity walking exercises (Milne et al., 2007; Rogers et al., 2007, 2009; Weathers et al., 2006; Windsor et al., 2009) and are more favourable of home-based, flexible programmes as opposed to facility based programmes (Karvinen et al., 2006, 2007; Rogers et al., 2007, 2009).

Pinto and colleagues (2005) implemented a home-based, moderate intensity walking intervention. They provided participants with instructions on how to exercise at moderate-intensity, how to monitor heart rate and also how to warm up and cool down before and after exercising for their intervention. Participants were asked to complete exercise walking logs and to wear pedometers whilst they were walking for exercise as part of the intervention only not for a set period of time throughout their typical day. They encouraged participants to exercise at moderate intensity at 55%-65% of their maximum heart rate including activities such as brisk walking. Participants were encouraged to walk at moderate intensity for 10 minutes on at least two days per week at the beginning of the intervention. As they progressed through the intervention this increased to 30 minutes 5 times a week in line with NICE recommended guidelines. Similarly, participants randomised to walking intervention by Mock et al. (2005) were asked to walk five to six times per week at moderate intensity at approximately 50-70% of maximum heart rate. Participants were asked to walk briskly for 15 minutes to begin with and this increased to 30 minutes as the intervention progressed. Yang et al. (2010) asked breast cancer patients receiving adjuvant chemotherapy to walk briskly three times per week for 12 weeks. Participants were asked to begin with 5 minutes warm up prior to 30 minutes of moderate intensity brisk walking for 30 minutes at 60-80% of maximum heart rate followed by 5 minutes cool down. Heart rate was monitored objectively by asking participants to wear a Polar heart rate monitor during exercise sessions and subjectively by asking them to monitor their heart rate. Alternatively, Holmes et al. (2005) and Holick et al. (2008) asked patients to subjectively rate their usual walking pace using a four point Likert scale. Participants were asked to rate their walking speed in miles per hour; for example, easy or casual pace was scored as 2mph whereas very brisk walking was scored as 4mph.

Evidence suggests that physical activity of moderate intensity has positive effects on psychosocial factors in breast cancer patients using a variety of activities. Campbell et al. (2005) found positive effects of a range of physical activities completed at moderate intensity and 60-75% of maximum heart rate, including walking, cycling, low-level aerobics and muscles strengthening circuits. Participants in their intervention group displayed significant improvements on measures of physical functioning and overall quality of life. In addition, Daley et al. (2007) found positive effects of moderate intensity aerobic exercise on overall quality of life. Previous studies implementing moderate intensity walking interventions have focused on the introduction of brisk walking to increase heart rate to between 50 and 80% of maximum heart rate (Pinto et al., 2005; Mock et al., 2005 and Yang et al., 2010) These studies have shown positive effects in improving physical fitness, fatigue, emotional distress and mood in breast cancer patients receiving adjuvant chemotherapy.

*Physical activity and cognitive functioning*

Physical activity has shown improvements in cognitive functioning in children and in the older generation as well as providing them with a sense of accomplishment, making them feel better about themselves, improving self-esteem and improving self-perception of body image (Department of Health 2004).

Aerobic physical activity has been demonstrated as having positive effects on cognition in healthy individuals, children and adolescents and within the older generation affected by dementia. A number of cross sectional studies using healthy adults have demonstrated the positive effects of aerobic activity on cognition (Christensen et al., 1996; Colcombe et al., 2004; Bixby et al., 2007; Deeny et al., 2008; Newson & Kemps, 2006; van Gelder et al., 2004 and Barnes et al., 2003). Colcombe et al. (2004) found positive effects of moderate intensity aerobic exercise on cognitive function. Healthy individuals within their trial began with aerobic activities and walking of 40-50% maximum heart rate increasing to 60-70% as the intervention progressed. The duration of exercise also increased, with participants beginning with 10-15 minutes of walking and increasing to 40-45 minutes by the end of the intervention. In addition, Bixby et al. (2007) found positive an association between executive function and exercise in healthy women aged between 65-92 years as measured by the Stroop test. Similarly, Weave at al. (2008) found higher levels of physical activity such as walking were associated with better cognitive performance in a global score of 6 tests including working memory, attention and verbal memory. Mean global cognitive scores of women walking at an easy pace for at least 1.5 hours a week were 0.06-0.07 units higher (of mean scores calculated from six neuropsychological tests) in comparison with women walking less than 40 minutes per week. Evidence as outlined above illustrates positive effects of easy-moderate levels of aerobic exercise and walking upon cognitive functioning in healthy adults. Taking this into account, it could be possible that moderate intensity physical activity may help to protect and maintain cognitive functioning in breast cancer patients.

Similarly, physical activity has not only shown positive results for cognition in children and adolescents but has also shown positive effects for increasing psychological well-being (Mutire & Parfitt, 1998). A meta-analysis including 44 studies, conducted by Sibley & Etnier, (2003) concluded that there is a significant positive relationship between physical activity and cognitive functioning in children. Furthermore, they found no significant difference in the types of physical activity performed by children who were aged between 4 and 18 years. Types of physical activity included within the meta-analysis which showed no significant difference included resistance/circuit training, PE programs, aerobic exercise including brisk walking and perceptual-motor training. Results from the meta-analysis Suggest any type of physical activity can ultimately benefit children’s cognitive performance. A range of cognitive abilities demonstrating significant positive effects were tested including: perceptual skills, developmental level, verbal skills and memory. Sibley and Etinier found a significant overall effect size of 0.32 indicating a positive association between physical activity and cognition for children. Effect size was calculated using Hedge’s g calculation to compare cognitive performance of the intervention groups with control groups. Groups that were exposed to physical activity, of all types, showed improvements in cognition equivalent to approximately half a standard deviation.

In addition, evidence also suggests positive effects of physical exercise in preventing severe cognitive dysfunctions such as dementia. Five out of thirteen studies evaluated in a review of the effects of physical exercise on cognition in the elderly (Clifford et al., 2009) found a reduced risk of Alzheimer’s in those who exercised often. A prospective analysis of risk factors for Alzheimer’s disease conducted by Lindsay et al. (2002) revealed physical activity as an important component in preventing the development of Alzheimer’s and many other conditions. In their large sample of 4,615 individuals aged over 65 years, they found regular physical activity reduced the risk of developing Alzheimer’s by 31% as screened for using the Modified Mini Mental State (Teng & Chui, 1987). Similarly, Sumic et al. (2007) revealed positive effects of physical activity in preventive cognitive impairment in a sample of 66 people with a mean age of 88.5 years. They revealed that women who exercised more than four hours per week had 88% less chance of developing cognitive impairments in comparison to those who exercised less than four hours a week (the intensity of exercise was not reported). Interestingly, these results were not reflected in the male population of the sample, suggesting that regular physical activity protects females more so than males against developing cognitive impairments later in life as tested using the Mini Mental State Examination and the Clinical Dementia Rating Scale (Morris et al., 1993).

The evidence discussed demonstrates promising results for physical activity acting as a protective mechanism in preventing cognitive impairment in healthy individuals, children and the elderly. In light of this evidence it is fair to hypothesise that moderate levels of physical activity may also help in protecting breast cancer patients against chemotherapy-induced cognitive impairment. Furthermore, the copious amount of research outlining the positive effects of physical activity in reducing emotional distress and improving self-esteem suggests physical activity may also act as mechanisms in improving cognitive functioning in breast cancer patients.

As outlined above, there is a host of evidence illustrating the benefits of physical activity rehabilitation for cancer patients. However, literature has not yet examined the effects of physical activity on cognitive functioning for those affected by cancer. Current studies implementing physical activity as a form of rehabilitation for breast cancer have focused upon the benefits it has in physical fitness, quality of life, fatigue and emotional distress. There is a need to explore the effects of physical activity upon cognitive functioning in breast cancer patients.

In summary, a small proportion of breast cancer patients demonstrate cognitive dysfunction in domains such as memory, attention, concentration and executive function which have been shown to be associated with baseline measures of fatigue, depression and emotional distress. Current interventions aimed at maintaining cognitive functioning have not been effective as they fail to account for emotional distress as a possible mechanism between chemotherapy and cognitive functioning. Therefore, there is a need to investigate emotional distress as a mechanism in cognitive interventions. In addition, literature suggests positive effects of moderate intensity physical activity for improving emotional distress, self-esteem and fatigue in breast cancer patients. Physical activity has also been shown to enhance cognitive functioning and prevent cognitive impairment in healthy adults, children and those affected by dementia. Therefore, the current study aims to manage emotional distress reported by breast cancer patients and reduce the cognitive demand it places on domains of memory, attention and executive function. The study will investigate this through the implementation of a home-based, moderate intensity walking exercise in comparison with usual care for a randomised sample of breast cancer patients receiving adjuvant chemotherapy.

**Research objectives/ Hypotheses**

The overall aim of the study is to implement a physical activity intervention to alleviate emotional and cognitive side effects experienced by those affected by breast cancer. Particular objectives are to:

1) Investigate the effectiveness of a home-based, moderate intensity walking intervention in increasing levels of self-esteem, psychological well-being and in reducing emotional distress

2) Investigate the effect of a home-based physical activity intervention on cognitive functioning in breast cancer patients receiving adjuvant chemotherapy

The study will test the following key hypotheses:

1. Breast cancer patients who receive the physical activity intervention will show improved performance on self-report measures of cognitive functioning compared with the control group.
2. Breast cancer patients who receive the physical activity intervention will report improved quality of life, higher self-esteem and lower emotional distress compared with the control group.
3. Enhanced cognitive performance in domains of memory, executive functioning and attention will be mediated by improved quality of life and self-esteem and lower levels of emotional distress following completion of the home-based moderated intensity walking intervention.

**Methodology**

**Design**

The intervention will be delivered to breast cancer patients receiving neo adjuvant and adjuvant chemotherapy in the oncology department of the Leicester Royal Infirmary Hospital. Upon recruitment all participants will be screened to ensure they meet the inclusion criteria to take part in the study. All those meeting the criteria will be randomised to either the intervention group or to the control group. Those who are randomised to the intervention group will receive usual care and the physical activity intervention. Patients randomised to the control group will receive usual care alone. However, following completion of the study, individuals randomised to the control group in the first instance will be provided with the physical activity intervention.

Following initial screening breast cancer patients who meet the criteria will be randomised to one of the following groups.

1. Intervention group (individuals randomised to the physical activity )
2. Control group (individuals randomised to receive usual care)

Data will be collected:

1. Before receiving chemotherapy (time 1)
2. After two cycles of chemotherapy and prior to the delivery of the intervention (time 2)
3. After 6 cycles of chemotherapy, following the completion of the intervention (time 3)

All data will be collected from January 2012 to December 2012.

**Sampling**

Breast cancer patients will be recruited from the Leicester Royal Infirmary Hospital. Patients who are receiving adjuvant or neo adjuvant chemotherapy and are considered fit to take part in moderate intensity exercise by their oncologist will be invited to participate in the study. Participants who meet the inclusion criteria for the study will be randomised to one of the two groups.

- Intervention group: this will consist of patients who will receive the physical activity intervention and usual care. Participants will be asked to complete a series of questionnaires and a battery of neuropsychological tests at three time points.
- Control group: this will consist of patients who will receive usual care. Participants will be asked to complete a series of questionnaires and a battery of neuropsychological tests at three time points.

Participants who are randomly assigned to the control group in the first instance will be given the opportunity to take part in the physical activity intervention, following the completion of the study.

- All participants will be asked to wear an accelerometer for 10 hours a day for 7 days at time 1 and 2. In line with previous findings, 7 days of continuous monitoring is recommended to assess habitual physical activity in adults and provides a trade-off between feasibility, reliability and acceptable participant burden (Corder, Brage & Ekelund 2007). In addition, participants in the intervention group only will be asked to wear a pedometer 10 hours a day for the duration of the 6 week intervention. Participants in the intervention group will be asked to wear pedometers to provide a measure of their step count. For this reason, individuals within the control group will not be provided with a pedometer. Participants within the intervention group will also be asked to subjectively rate the intensity of their exercise during the six week intervention period.

**Inclusion criteria for participation**

Participants will include females with a primary diagnosis of stage I-III breast cancer. Patients who are receiving adjuvant or neo adjuvant chemotherapy will be invited to take part within the study. Only participants over the age of 18 will be asked to take part in the study.

**Exclusion criteria for participation**

Patients with secondary or metastatic diagnoses will not be included within this study. Patients with a history of major psychiatric disorders which are likely to lead to cognitive deficits will also be excluded from taking part within this study. Patients who exceed 150 minutes of moderate intensity exercise a week will not be included within the study. If patients are physically active, it is assumed that they will be receiving its benefits.

**Overview of the intervention**

*Physical activity element of intervention*

- Participants will be provided with an intervention booklet. The booklet will include: guidance and recommendations to ensure adherence to the intervention, tips and encouragement outlining the benefits of walking, and a diary to keep a log of walking duration and intensity. Participants will also be asked to keep a log of adverse events such as dizzy spells, sore leg which may affect adherence to recommended levels of exercise.
- Patients within the intervention group will be asked to take part in a home-based, moderate intensity walking intervention for the duration of the study. Moderate intensity physical activity as defined by the Department of Health (2004) refers to an individual usually experiencing “increased breathing rate, an increase in heart rate to the level where the pulse can be felt and is usually accompanied by a feeling of increased warmth and maybe sweating”. Intensity of walking will be subjectively measured using the Talk Test (Persinger, 2004) and the Borg Rating of Perceived Exertion Scale (Borg, 1998).
- In line with previous studies and recommended guidelines (Department of Health, 2004), participants will be asked to exercise for 30 minutes five times a week at moderate intensity.
- Participants will be asked to steadily increase walking duration as they progress through the intervention. Initially participants will be asked to walk briskly for 10 minutes and steadily increase the duration of walking to 30 minutes as the intervention progresses. Participants will be advised to steadily increase their walking duration throughout the 12 week intervention period. They will be advised to increase walking by 2-3 minutes a week aiming to reach 30minute bouts towards the end of the intervention.
- Participants will be asked to keep a weekly diary outlining the duration of walking exercises and a record of perceived exertion.
- Participants within the control group and the physically active group will also be asked to keep a physical activity log.

**Measures**

*Measures of physical activity*:

- *Accelerometers* will be worn by all participants for 10 hours a day for 7 days at baseline and post-intervention to assess habitual physical activity. Accelerometers are motion sensors which detect accelerations produced by the human body (Welk, 2002). Welk (2002) defines acceleration as the rate of change in velocity over a given time. Accelerometers can be clipped to clothing or worn on a belt above the hip and measure the frequency, intensity and duration of physical activity assessed through body movement.
- *Pedometers* will be worn by participants randomised to the intervention group for 10 hours a day for the duration of the 12 week intervention. Pedometers can be attached to participants clothing above the hip and record frequency of steps taken. Participants will be asked to make a daily note of steps taken before resetting the device. The pedometers will act as a form of motivation encouraging participants to increase their step count. For this reason individuals within the control group will not be provided with pedometers.
- *Borg Rating of Perceived Exertion Scale* (Borg, 1998) will be completed by participants in the intervention group to measure the intensity of walking exercises. The scale asks participants to rate how hard they feel their bodies are working based on the physical sensations they experience including increased heart rate, breathing rate and sweating. Exertion is measured on a rating scale with ‘6’ indicating ‘no exertion at all’ and ‘20’ indicating ‘maximal exertion’. Moderate intensity exercise is rated between 12 and 14 on Borg’s scale.
- *The Talk Test (*Persinger, 2004) will be used by participants within the intervention group as a guide to monitor the intensity and pace of their walking. The informal subjective measure allows individuals to judge their own intensity with the understanding that if they are carrying out moderate intensity walking they should still be able to maintain a conversation but not sing. Whereas carrying out vigorous intensity exercise will prevent individuals from speaking more than a couple of words.

*Subjective:*

The following measures will be completed at three time points; pre-chemotherapy (time 1), pre intervention (time 2) and post-intervention (time 3).

- *Anxiety and depression* will be measured using the Hospital Anxiety and Depression Scale (Sigmund 1983). The ten item scale will be used to determine the levels of anxiety and depression that a patient is experiencing. Responses to the items are measured on a four-point Likert scale and take approximately 5-10 minutes to complete.
- *Fatigue* will be measured using The Functional Assessment of Cancer therapy-Fatigue (Yellen et al. 1997). The 13 item fatigue scale asks participants to rate the way they have felt in the last 7 days using a 5 point Likert scale (0= not at all to 4=very much).
- *Self-esteem* will be measured using the Self-Esteem Scale (Rosenberg 1965). The rating scale requires participants to indicate how they feel in relation to a series of 10 statements. Participants are asked to rate levels of self-esteem on a four point Likert scale (1= strongly agree to 4=strongly disagree).
- *Emotional Distress* will be measured using the shortened version of Profile of Mood States (Schacham, 1983). The tool is commonly used within the cancer population and requires participants rate 37 adjectives on a 5 point scale ranging from ‘not at all’ to ‘extremely’.
- *Subjective cognitive functioning* will be measured using the Cognitive Failures Questionnaire, (Broadbent, Cooper, FitzGerald & Parkes, 1982). The 25 item scale will be used to look at minor mistakes made across a one month time frame. Responses are measured on a five-point Likert scale and take approximately 10-15 minutes to complete.
- *Physical activity* will be measured using the General Practice Physical Activity Questionnaire. The 3 item questionnaire asks participants how much physical activity they have carried out during the last week at work and leisure. The questionnaire also asks participants to state their usual walking pace.
- *Intention to exercise* will be measured using the Physical Activity Stages of Change Questionnaire (SOC) (Marcus & Simkin, 1993). The SOC is a 4-item tool based on the stage of change model (Prochaska & DiClemente; 1984), measuring motivational readiness to adopt physical activity behaviour. The stages of change include: precontemplation (not considering changing one’s behaviour), contemplation (thinking about change), preparation (making definite plans to change), action (here the individual has changes their behaviour) and finally maintenance (working to prevent relapse and consolidate the gains made) or relapse.

*Objective:*

The following neuropsychological tests will be completed by participants before beginning chemotherapy (time 1), before beginning the intervention (time 2) and following the completion of the intervention (time 3).

- Executive function will be measured using the Stroop test (Stroop, 1935). The test has commonly been used within previous studies illustrating cognitive dysfunction in chemotherapy patients (Jansen et al., 2008; Jenkins et al., 2006; Mehlsen et al., 2009; Shilling et al., 2005 and van Dam et al., 1998). The test requires participants to attend to a target whilst simultaneously suppressing a habitual response in favour of a less familiar one.

The computer based test will be demonstrated using colour names: two sets of different colour names (red, green, blue etc) will be presented on screen. One will be written in the appropriate colour for the word, while the other set are written in a different colour (e.g. the word ‘red’ written in blue ink). On being asked to identify the colours in each list, subjects take longer to process the information which contains discrepant information. Reading the colour name occurs as an automatic cognitive sub-routine, which interferes with the recognition of the colour itself. The test will take approximately 5 minutes to administer.

- Working memory will be tested using forwards and backwards digit span as used in the Wechsler Adult Intelligence Scale-III (The Psychological Corporation, 1997). Digit span is commonly used to demonstrate chemotherapy induced cognitive decline in breast cancer patients (Collins et al., 2009; Jenkins et al., 2006; Mehlsen et al., 2009; Schagen et al., 1999; Shilling et al., 2005; van Dam et al., 1998 and Wefel et al., 2004). A meta- analysis of the sensitivity of neuropsychological tests used to detect chemotherapy induced cognitive impairment in breast cancer patients (Jansen et al. 2007) found that digit span produced the largest effect size out of all the tests they reviewed. In addition, forwards and backwards digit span is not affected by ceiling effects.

Each task is made up of six pairs of numbers which are read aloud by the researcher. Digits forwards require participants to repeat number sequences in the same order as they are presented. Whereas, digits backwards requires participants to repeat the number sequence in reverse order. When a sequence is repeated correctly, the researcher will continue to read aloud a longer sequence of numbers. This is repeated until the participant fails to repeat a sequence correctly. Therefore, the higher the score the better the performance.

- Sustained attention will be measured using a continuous performance vigilance task created by the researchers using E-Prime and the Sustained Attention to Response Task (SART), (Robertson et al. 1997).

The continuous performance vigilance task consists of a ‘simple’ and a ‘complex’ task as adapted from Altena et al. (2008). The ‘simple’ task requires participants to press the left button of the mouse, with their dominant hand, each time they see one of the 110 asterisks sequentially appear on the screen. The target will appear in the same location but in timed intervals between 1 and 10seconds. The ‘complex’ task requires participants to respond as quickly and accurately as possible to target letters ‘d’ and ‘p’ which are demonstrated amongst 10 distracters. Target letters are presented randomly between 0.5 and 5 seconds. The continuous performance vigilance task has been selected due to its previous success in detecting sustained attention difficulties in breast cancer patients. Ahles et al. (2008) found significantly lower reaction times as measured by the continuous performance vigilance task in individuals diagnosed with stage I-III breast cancer.

The SART is a computer based task which presents participants with sequences of digits from 1-9 in random order at a rhythmic rate of one every 1.9 seconds. The visually presented stimuli may be displayed up to 25 times and participants are required to respond to each digit except from the number ‘3’. Participants are asked to press the same response key each time which rapidly becomes an automatic response. However, participants are required to resist this automatic response when they are presented with the number ‘3’.

- Visualspatial skills will be measured using the WAIS Block Design subset as used in the Wechsler Adult Intelligence Scale-III (The Psychological Corporation, 1997). This test has been selected as it demonstrated significant moderate effect sizes of sensitivity in determining chemotherapy induced cognitive decline in breast cancer patients in a meta-analysis conducted by Jansen and colleagues in 2007.

WAIS block design subset requires participants to arrange a set of blocks in the same pattern as that demonstrated by the researcher. Materials include block with all white sides, blocks with all red sides and block with a combination of both white and red sides. The time taken to complete block design patterns is compared to a normative sample.

Demographic and cancer diagnosis data will be collected from all participants at time one.

Additional post-test measures will be included after time 1 on the following:

- Change in status: participants will be asked if they have experienced changes regarding the stage of their cancer and the type of treatment they are receiving. They will also be asked to state changes in their personal lives. Participants will be asked these questions at time 3 (post intervention).
- Adherence to the intervention: Participants will be asked to keep a log of the duration and frequency of walking completed each week for the duration of the 12 week intervention.

**Procedure**

- Breast cancer patients undergoing adjuvant or neo adjuvant chemotherapy will be recruited following appointments with oncologists and nursing staff at the Leicester Royal Infirmary. Potential participants who express an interest in the study will be provided with an information sheet and will be asked to provide a contact telephone number and email address.

- Approximately 1 week (at least 48 hours) following the initial recruitment stage, the research team will contact patients to ask if they are interested in taking part in the study. Patients who are interested will be asked to complete a recruitment questionnaire to ensure they meet the studies inclusion criteria.

- Participants who meet the criteria and wish to take part in the study will be asked to provide informed consent following which the research team will arrange a convenient time for the individual to complete a series of questionnaires and a battery of neuropsychological measures (time 1) before they begin chemotherapy. At this stage, all participants will be asked to wear an accelerometer for 10 hours a day for 7 days.

- Six weeks later and after 2 cycles of chemotherapy, patients will meet with the researcher to complete pre intervention measures (time 2). At this stage participants will be randomised to either the intervention or control group. Those who are randomised to the intervention group will receive pedometers and guidance booklets for the intervention. Participants will be provided with a booklet outlining the amount of walking they should take part in alongside guidance and tips on how to incorporate this into their daily routines.

- Participants will be asked to commit to a minimum of 150 minutes of moderate intensity walking per week for the duration of the 12 week intervention, in line with recommended guidelines outlined by the Department of Health. Participants will be told how to monitor their own intensity levels and will be encouraged to aim to walk at moderate levels of intensity. Participants in the intervention group will also be asked to wear a pedometer for 10 hours a day for the duration of the 12 week intervention and keep a log of steps taken per day.

However, the researcher will make it clear that participants should only carry out exercises within their own limits but should aim to reach their personally set goals.

-Walking schedules will be self-prescribed by participants but the researcher will recommend they begin by completing 10minutes of walking at any one time and then steadily increase the duration of walking to 30minutes.

-The research team will emphasise the importance of looking after their bodies and not committing to more walking than their bodies are capable of. The research team will gain consent from each participant to contact the consultant or cancer nurse should they be concerned about the patient’s health/well-being.

-Participants will be asked to keep a log of their walking for the duration of the 12 week study.

-Post-intervention (week 19), all participants will be asked to wear the accelerometer for a further 10 hours a day for 7 days. In addition, they will be asked to complete a series of questionnaires and a battery of neuropsychological tests. Measures will include objective and subjective measures of cognitive functioning and self-report measures of emotional well-being.

-Testing will take place at the Leicester Royal Infirmary Hospital. The researcher will endeavour to arrange tests to be completed whilst patients are waiting for other appointments. However, if more convenient for the patient, the researcher can meet with them within their homes or at Loughborough University.

-Neuropsychological tests and questionnaires should be completed within close proximity of one another. However, patients will be given the option to take the questionnaire away with them and complete it at their leisure within their own homes.

- A summary of the results of the study will be available for participants 12-18 months following the completion of the study.

Recruitment Screening

Baseline Testing (T1)

Acceleration measured for 7days

Pre-intervention testing (T2)

Randomisation

Intervention group

Control group

Usual care alone

12 week home based moderate intensity walking intervention plus usual care

Post-intervention testing (T3)

Acceleration measured for 7days

*Flow diagram outlining participants journey through the study*

**Statistical Analyses**

The data will be entered into Predictive Analytics SoftWare (PASW) and will be analysed using repeated measures ANOVA with significant main effects explored further using bonferonni-corrected comparisons.

Multiple regressions will also be used to investigate the predictive value of some of the measures towards primary outcome variables (controlling for treatment type, cancer stage, education level and socio-economic status).

**Ethics**

*Recruitment:*

This study will involve women who have recently been diagnosed with breast cancer and are due to undergo treatment. The researcher acknowledges that such individuals may be sensitive and vulnerable. Therefore, the recruitment and assessment procedures have been tailored to minimise stress and discomfort. All participants will be provided with a clearly written and accessible information sheet as to the purpose of this study. Patients will have time to ask questions about the study during the briefing meeting. Participants will be made aware that they are under no obligation to take part in the study. Each participant will be informed that they can withdraw from the study without having to give a reason at any point and without any adverse consequences, particularly in relation to the use of the facilities and services at provided at the Leicester Royal Infirmary. Full informed written consent will be obtained prior to any data collection. Patients who are not randomised to the intervention group in the first instance will be given the opportunity to take part in the intervention following the completion of the study. Therefore, those individuals who form group two will not be disadvantaged.

*Data collection:*

The research team will not be delivering the intervention directly as participants will be developing their own walking plans. Similarly researchers will be facilitating the peer support group and will not be leading the content of discussion. The researcher will merely ensure that participants feel comfortable in their surroundings. Diagnosis and treatment-related data for each group will be obtained through the Leicester Royal Infirmary. Written permission will be sought from each individual prior to accessing this data. At the end of the study period, all participants will be debriefed regarding the nature of the research and will be provided with a summary of the key findings.

*Data storage and Confidentiality:*

All data collected will be treated with confidentiality stored securely and will be accessible only to the researchers and will not be revealed to a third party. All participant personal details will be kept separately and securely under conditions of strict confidentiality from the study data itself as required by the law in the Data Protection Act 1998. Anonymity of all participants will be ensured. The British Psychological Society’s Code of Conduct and Ethical Guidelines for research will be adhered to at all times. Participants will be given a unique study code reference number (identifier) which will be used rather than the participant name e.g. on questionnaire, during data entry and analysis. Only anonymous data will be reported.

*References:*

Ahles, T. A., Saykin, A. J., Furstenberg, C. T., Cole, B., Mott, L. A., Skalla, K., et al. (2002).Neuropsychologic impact of standard-dose systemic chemotherapy in long-term survivors of breast cancer and lymphoma. *Journal of Clinical Oncology, 20*(2), 485-493

Arndt, V., Merx, H., Stegmaier, C., Ziegler, H., Brenner, H. (2005). Persistance of Restictions in Quality of Life From the First to the Third Year After Diagnosis In Women With Breast Cancer. *Journal of Clinical Oncology, 23*, 4945-4953.

Barnes, D.E., Yaffe, K., Satariano, W.A. & Tager, I.B. (2003). A longitudinal study of cardiorespiratory fitness and cognitive function in healthy older adults. *Journal of the American Geriatrics Society, 51* (4), 459-465.

Basen-Engquist, K., Taylor, C.L.C., Rosenblum, C., Smith, M.A., Shinn, E.H., Greisinger, A., Gregg, X., Massey, P., Valero, V., Rivera, E. (2006). Randomized pilot test of a lifestyle physical activity intervention for breast cancer survivors*. Patient Education and Councelling, 64,* 225-234.

Bender, C. M., Sereika, S. M., Berga, S. L., Vogel, V. G., Brufsky, A. M., & Paraska, K. K., et al. (2006). Cognitive impairment associated with adjuvant therapy in breast cancer. *Psycho-Oncology, 15*(5), 422-430.

Berglund, G., Bolund, C., & Fornander, T. (1991). Late effects of adjuvant chemotherapy and postoperative radiotherapy on quality of life among breast cancer patients. *European Journal of Cancer, 27*, 1075-1081.

Blows, W.T. (2005). *The biological basis of nursing: Cancer.* London and New York: Routedge Taylor & Francis Group.

Brezden, C B., Phillips, K-A., Abdolell, M., Bunston, T., & Tannock, I. F. (2000). Cognitive function in breast cancer patients receiving adjuvant chemotherapy. *Journal of Clinical Oncology, 18*(14), 2695-2701.

Bixby, W.R., Spalding, T.W., Haufler, A.J., Deeny, S.P., Mahlow, P.T., Zimmerman, J.B., & Hatfield, B.D. (2007). The unique relation of physical activity to executive function in older men and women. *Medicine & science In Sports & Exercise. 39* (8), 1408-1416.

Broadbent, D. E., Cooper, P. F., FitzGerald, P., & Parkes, K. R. (1982). The Cognitive Failures Questionnaire (CFQ) and its correlates. *British Journal of Clinical Psychology, 21*, 1-16.

Brezden, C B., Phillips, K-A., Abdolell, M., Bunston, T., & Tannock, I. F. (2000). Cognitive function in breast cancer patients receiving adjuvant chemotherapy. *Journal of Clinical Oncology, 18*(14), 2695-2701.

Buettner, C., Kroenke, C.H., Phillips, R.S., Davis, R.B., Eisenberg, D.M., Holmes, M.D. (2006) Correlates of use of different types of complementary and alternative medicine by breast cancer survivors in the nurses’ health study. *Breast Cancer Research Treatment*, *100,* 219–27

Campbell, A., Mutire, N., Whytee, F, et al. (2005). A pilot study of a supervised group exercise programme as a rehabilitation treatment for women with breast cancer receiving adjuvant chemotherapy. European Journal of Oncology Nursing, 9, 56-63.

Cancer Research UK. (2008b). *Breast cancer briefsheet.* Retrieved December 4, 2008, from http://publications.cancerresearchuk.org/WebRoot/crukstoredb/CRUK_PDFs/CRBSBRC08.pdf

Christensen, H., Korten, A, A., Jorm, A.F., Henderson, A.S., Scott, R. & Mackinnon, A.J. (1996). Activity levels and cognitive functioning in an elderly community sample*. Age and Ageing, 25*, 72-80.

Clifford, A., Bandelow, S., Hogervorst, E. (2009). The Effects of Physical Exercise on Cognitive Function in the Elderly: A Review. *Handbook of Cognitive Aging,* 4.

Cohen, S., Kamarck, T., Mermelstein, R. (1983). A global measure of perceived stress, *Journal of Health and Social Behaviour*, *24*, 385-96.

Cohen, L., Warneke, C., Fouladi, R.T., Rodriguez, M.A., Chaoul-Reich, A. (2004) Psychological adjustment and sleep quality in a randomized trial of the effects of a Tibetan yoga intervention in patients with lymphoma. *Cancer, 15,* 2253–60.

Colcombe, S.J., Kramer, A.F., Erickson, K.I., Scalf, P., McAuley, E., Cohen, N.J., Webb, A., Jerome, G.J., Marquez, D.X. & Elavsky, S. (2004). Cardiovascular fitness, cortical plasticity and aging. *PNAS, 101 (9)*, 3316-3321.

Courneya, K.S., Mackey, J.R., Bell, G.J., Jones, L.W., Field, C.J., & Fairey, A.S. (2003). Randomized controlled trial of exercise training in post-menopausal breast cancer survivors: cardiopulmonary and quality of life outcomes. *Journal of Clinical Oncology, 21*, 1660-1668.

Courneya, K.S., Segal, R.J., Gelmon, K., Reid, R.D., Mackey, J.R., Friedenrich, C.M., Proulx, C., lane, K., Ladha, A.B., Vallance, J.K., Liu, Q., Yasui, Y& McKenzie, D.C. (2007). Six-Month Follow-up of Patient-Rated Outcomes in a Randomised Controlled Trial of Exercise Training during Breast Cancer Chemotherapy*. Cancer, Epidemiology, Biomarkers & Prevention, 16,* 2572-2578.

Daley, A.J., Crank, H., Saxton, J.M. et al (2007). Randomised trial of exercise therapy in women treated for breast cancer*. Journal of Clinical Oncology, 25*, 1713-1721.

Deeny, S.P., Poeppel, D., Zimmerman, J.B., Roth, S.M., Brandauer, J., Witkowski, S., Hearn, J.W., Ludlow, A.T., Contrearas-Vidal, J.L., Brandt, J. & Hatfield, B.D. (2008). Exercise, APOE, and working memory: MEG and behavioural evidence for benefit of exercise in epsilon4 carriers. *Biological Psychology, 78*, 179-187.

Falleti, M.G., Sanfilippo, A., Maruff, P., Weih, L.A., & Phillips, K.A, (2005). The nature and severity of cognitive impairment associated with adjuvant chemotherapy in women with breast cancer: A meta-analysis of the current literature. *Brain and Cognition*, 59, 60-70.

Fillion, L., Gangan, P., Leblond, F., Gelinas, C., Savard, J., Dupuis, R., Duval, K., & Larochelle, M. (2008). A Brief Intervention for fatigue Management in Breast Cancer Survivors. Cancer Nursing, 31, 145-159.

Ferguson, R.J., Ahles, T.A., Saykin, A.J., McDonald, B.C., Furstenberg, C.T., Cole, B.F & Mott, L.A. (2007). Cognitive-behavioural management of chemotherapy-related cognitive change. *Psycho-Oncology, 16*, 772-777.

Jansen, C.E., Miaskowski, C.A., Dodd, M.J., & Dowling, G.A. (2007). A meta-analysis of the sensitivity of various neuropsychological tests used to detect chemotherapy-induced cognitive impairment in patients with breast cancer. Oncology Nursing Forum, 34 (5), 997-1005.

Kim, S.H., Son, B.H., Hwang, S.Y. et al (2008). Fatigue and depression in disease-free breast cancer survivors: prevalence, correlates, and association with quality of life. *Journal of Pain Symptom Manage, 35* (6) 644-655.

Kumar, V., Cotran, R.S., & Robins, S.L. (1997). *Basic pathology* (6^th^ ed.): W.B. Saunders Company.

Lazarus, R.S., & Folkman, S. (1984). Stress, Appraisal and Coping. New York: Springer

Lev, E.L., Daley, K.M., Conner, N.E., Reith, M., Fernandez, C., Owen, S.V. (2001) An intervention to Increase Quality of Life and Self-Care Self-Efficacy and Decrease Symptoms in Breast Cancer Patients. *Research and Theory for Nursing Practice, 15*, 277-294.

Lindsay, J., Laurin, D., Verreault, R., Hebert, R., Helliwell, B., Hill, B.B., McDowell, I. (2002). Risk Factors ffor Alzheimer’s Disease: A Prospective Analysis from the Canadian Study of Health and Aging. *American Journal of Epidemiology*, 6, 445-453.

Loiselle, C.G., Edgar,l., Batist, G., Lue,L., Lauzier, S. (2010) The impact of a multimedia informational intervention on psychosocial adjustment among individuals with newly diagnosed breast or prostate cancer: A feasibility study. *Patient Education and Counseling* 80 48–55

Manos, D., Sebastian, J., Mateos, N., & Bueno, M.J. (2009) Results of a multi-componential psychosocial intervention programme for women with early-stage breast cancer in Spain: quality of life and mental adjustment.  *European Journal of Cancer Care, 18*, 295–305.

Marcus, A., Garrett, K.M., Cella, D., Wenzel, L., , Brady, M.J.,Fairclough, D.,

Pate-Willig, M., Barnes, D., Emsbo,P., Kluhsman, B.C., Crane, L., Sedlacek, S., and J. Flynn, P.J. (2010) Can telephone counseling post-treatment improve psychosocial outcomes among early stage breast cancer survivors? *Psycho-Oncology* 19: 923–932

Maryam, A., Fazlollah, A., Eesa, M., Ebrahim, H. And Abbas, VF. (2010) The effect of designed exercise programme on quality of life in women with breast cancer receiving chemotherapy. *Scandinavian journal of caring sciences, 24,* 251–258.

McNeeley, M.L., Campbell, K.L., Rowe, B.H., Klassen, T.P., Mackey, J.R., Courneya, K.S. (2006). Effects of exercise on breast cancer patients and survivors: a systematic review and meta-analysis,*CMAJ 175*, 34-41.

Mehlsen, m., Pedersen, A.D., Jensen, A.B., & Zachariae, R. (2009). No indications of cognitive side-effects in a prospective study of breast cancer patients receiving adjuvant chemotherapy. *Psycho-Oncology, 18*, 248-257.

Merluzzi, T.V., Nairn, R.C., Hegde, K., Martinez Sanchez, M.A., Dunn, L. Self-efficacy for coping with cancer: revision of the Cancer Behavior Inventory (version 2.0) Psychooncology, 10, 206–217.

Moadel, A.B., Shah, C., Wylie-Rosett, J., Harris, M.S., Patel, S.R., Hall, C.B. (2007) Randomized controlled trial of yoga among a multiethnic sample of breast cancer patients: effects on quality of life. *Journal of Clinical Oncology, 28,* 4387–95.

Mock, V., Frangakis, C., Davidson, N.E., Ropka, M.E., Pickett, M., Poniatowski, B., Stewart, K.J., Cameron, L., Zawacki, K., Podewils, L.J., Cohen, G & mcCorkle. (2005). Exercise manages fatigue during breast cancer treatment: a randomised controlled trial. Psycho-Oncology, 14, 464-477.

Moorey, S., Frampton, M., Greer, S. (2003).The cancer coping questionnaire: a self-rating scale for measuring the impact of adjuvant psychological therapy on coping behaviour. *Psycho-Oncology, 12*, 331-344.

Mutrie, N., Campbell, A., Whyte, F, et al. (2007). Benefits of supervised group exercise program for women being treated for early stage breast cancer: pragmatic randomised controlled trial. *British Medical Journal, 334*, 517-523.

Mystakidou, K., Parpa, E., Tsilika, E., Gogou, P., Panagiotou, I., Galanos, A., Kouvaris, I., Gouliamos, A. (2010). Self-Efficacy, Depression, and Physical Distress in Males and Females With Cancer. *American Journal of Hospice and Palliative Medicine, 000*, 1-8

Newson, R.S & Kemps, E.B. (2006). Cardiorespiratory fitness as a predictor of successful cognitive aging. *Journal of Clinical and Experimental Neuropsychology, 2*8, 949-967.

Percorino, L. (2008). *Molecular biology of cancer: Mechanisms, targets and therapeutics* (2^nd^ ed.). Oxford: Oxford University Press.

Pinto, B.M., Frierson, G.m., Rabin, C., Trunzo, J.J., Marcus, B.H. (2005). Home-based physical activity intervention for breast cancer patients. Journal of Clinical Oncology, 23 (15) 3577-3587.

Pinto, B.M., Slark, M.M., Maruyama, N.C., Feder, S.I. (2003). Psychological and fitness changes associated with exercise participation among women with breast cancer. *Psych-Oncology, 12*, 118-126.

Poppelreuter, M., Weis, J., Bartsch, H.H. (2009). Effects of Specific Neuropsychological Training Programs for Breast Cancer Patients After Adjuvant Chemotherapy. *Journal of Psychological Oncology, 27*, 274-296.

Pullens, M.J.J., De Vries, J., & Roukema, J.A. (2010). Subjective cognitive dysfunction in breast cancer patients: a systematic review. *Psycho-Oncology, 19*, 1127-1138.

Schagen, S.B., van Dam, F.S., Muller, M.J., Boogerd, W., Lindeboom,J., & Bruning, P.F. (1999). Cognitive deficits after postoperative adjuvant chemotherapy for breast cancer carcinoma. *Cancer, 85* (3), 640-650.

Schou, I., Ekeberg,O., Karesen, R. and Sorensen, E. (2008) Psychosocial intervention as a component of routine breast cancer care*-*who participates and does it help? *Psycho-Oncology*, *17,* 716–720

Scheier, M.F., & Carver, C.S (1985). Optimism, coping, and health: Assessment and implications of generalized outcome expectancies. *Health Psychology,* *4,* 219-247.

Segal, R., Evans, W., Johnson, D., Smith, J., Colleta, S., Gayton, J., Wella, W.S.G., Reid, R. (2001) Structured exercise improves physical functioning in woment with stage I and II breast cancer: Results of randomised controlled trial. Ournal of Clinical Oncology,19, (3) 657-65.

Shapiro, S.L., Lopez, A.M., Schwartz, G.E., et al. (2001). Quality of life and breast cancer: relationship to psychosocial variables. *Journal of Clinical Psychology, 4*, 501-519.

Skarstein, J., Aass, N., Fossa, S., Skovlund, E., Dahl, A. (2000) Anxiety and depression in cancer patients: relation between the Hospital Anxiety and Depression Scale and the European Organization for Research and Treatment of Cancer Core Quality of Life Questionnaire. *J Psychosom Res,49*, 27–34.

Speca, M., Carlson, L.E., Goodey, E., Angen, M., (2000). A randomized, wait-list controlled clinical trial: the effect of a mindfulness meditation based stress reduction program on mood and symptoms of stress in cancer outpatients. *Psychosom. Med, 62*, 613–622.

Speilberger, C.D., Gorsuch, R.L., Lushene, R. (1970). Manual for the State-Trait Personality Inventory*: STAI*. Palo Alto, CA: *Consulting Psychologists Press*.

Stevinson, C., Lawlor, D.A., & Fox, K.R. (2004). Exercise interventions for cancer patients: systematice review of controlled trials. *Cancer Causes and Control, 15*, 1035-1056.

Stroop, J.R. (1935). Studies of inference in serial verbal reactions. *Journal of Experimental Psychology, 18,* 643-662.

Sumic, A., Michael, Y.L., Carlson, N.E., Howieson, D.B & Kaye, J.A. (2007). Physical activity and the risk of dementia in oldest old. *Journal of Aging and Health, 19* (2), 242-259.

Raghavendra, R.M., Nagarathna, R., Nagendra, H.R., Gopinath, K.S., Srinath, B.S., Ravi, B.D. (2007) Effects of an integrated yoga programme on chemotherapy induced nausea and emesis in breast cancer patients. *European Journal of Cancer Care, 16,* 462–74.

Roob, C., Haley, W.E., Balducci, L., Extermann, M., Perkins, E.A., Small, B.J., Mortimer, J. (2007). Impact of breast cancer survivorship on quality of life in older women. *Critical Reviews in Oncology/ Hematology*, *62*, 84-91.

Rosenbaum, E., Gautier, H., Fobair, P., Neri, E., Festa, B., Hawn, M. (2004) Cancer supportive care, improving the quality of life for cancer patients. A program evaluation report. *Support Care Cancer*, *12*, 293–301.

Tchen, N., Juffs., H.G., Downie, F.P., Qi-Long, Y., Hanzian, H., Chemerynsky, L., et al (2003). Cognitive function, fatigue and menopausal symptoms in women receiving adjuvant chemotherapy for breast cancer. *Journal of Clinical Oncology*, 21, 4175-4183.

Vadiraja, H.S., Raghavendra Rao,M., Raghuram Nagarathna, R.,Nagendra, H.R., Rekha, M., Vanitha, N., Gopinath, K.S., Srinath, B.S., Vishweshwara, M.S.,. Madhavi, Y.S, Ajaikumar, B.S., Bilimagga, S.R. and Rao, N. (2009) Effects of yoga program on quality of life and affect in early breast cancer patients undergoing adjuvant radiotherapy: A randomized controlled trial. *Complementary Therapies in Medicine*, *17,* 274—280

van Dam, F.s., Schagen, S.B., Muller, M.J., Boogerd, W, E.v.d., Fortuyn, M.E.D., et al. (1998). Impairment of cognitive function in women receiving adjuvant treatment for high-risk breast cancer: High-dose versus standard dose-chemotherapy. *Journal of the National Cancer Institute, 90*, 210-218.

Van Gelder, B.M., Tijhaus, M.A., Kalmijn, S., Giampaoli, S., Nissinen, A. & Kromhout, D. (2004). Physical activity in relation to cognitive decline in elderly men: the FINE study. *Neurology, 63* (12), 2316-2321.

VonEssen. L., Larsson, F., Oberg, K., Sjoden, P., (2002). Satisfaction with care: associations with health-related quality of life and psychosocial function among Swedish patients with endocrine gastrointestinal tumours. *Eur J Cancer Care, 11,* 91–99.

Voogt, E., Van Der Heide, A., Van Leeuwen, A.F., Visser, A.P., Cleiren, M.P., Passchier, L., Vand Der Mass, P.J. (2005) Positive and negative affect after diagnosis of advanced breast cancer. *Psychooncology,* 4, 262-73

Watson, D., Clark, L. A., Tellegen, A. (1988b). Development and validation of brief measures of positive and negative affect: The PANAS Scales. *Journal of Personality and Social Psychology*, *47*, 1063–1070

Watson, M., Greer, S., Young, J., Inayat, G., Burgess, C., Robertson, B. (1988) Development of a questionnaire measurement of adjustment: the MAC scale, *Psychological Medicine, 18,* 203-9.

Wechler, D. (1955). *WAIS manual*. New York: The Psychological Corporation.

Wefel, J.S., Lenzi, R., Theriault, R.L., Davis, R.N., & Meyers, C.A. (2004). The cognitive sequelae of non-central nervous system cancer and cancer therapy*. Neuropsychological Reviews,* 18, 121-131.

Weinberg, R.A. (2007). *The biology of cancer*. New York: Garland Science.

Weis, J., Poppelreuter, M., & Bartsch, H.H. (2009). Cognitive deficits as long-term side-effects of adjuvant therapy in breast cancer patients: ‘subjective’ complaints and ‘objective’ neuropsychological test results. *Psycho-Oncology, 18*, 775-782.

Yang, C., Tsai, J.,Huang, Y., & Lin, C. (2010). Effects of a home-based walking program on perceived symptom and mood status in postoperative breast cancer women receiving adjuvant chemotherapy. *Journal of Advanced Nursing, 67* (1), 158-168.

Yellen, S.B., Cella, D.F., Webster, K., Blenowski, C., & Kaplan, E. (1997). Measuring fatigue and other anemia-related symptoms with the Functional Assessment of Cancer Therapy (FACT) measuring system. *Journal of Pain and Symptom Management,* 13 (2), 63-74.,

Zenger, M., Glaesmaer,H., Hockel,M., Hinz, A. (2010). Pessimism Predicts Anxiety, Depression and Quality of Life in Female Cancer Patients. *Japanese Journal of Clinical Oncology*,40.

Zigmond, A.S., & Snaith, R.P. (1983). The Hospital Anxiety and Depresion Scale*. Acta Pschiatrica Scandinavic*a, 67, 361-370.
